# Multidrug-resistant tuberculosis surveillance and cascade of care in Madagascar: a five-year (2012–2017) retrospective study

**DOI:** 10.1186/s12916-020-01626-6

**Published:** 2020-06-30

**Authors:** Astrid M. Knoblauch, Simon Grandjean Lapierre, Daniella Randriamanana, Mamy Serge Raherison, Andrianantenaina Rakotoson, Bienvenue Solofomandimby Raholijaona, Masiarivony Ravaoarimanga, Pascaline Elisabeth Ravololonandriana, Marie-Sylvianne Rabodoarivelo, Orelys Ratsirahonana, Fanjasoa Rakotomanana, Turibio Razafindranaivo, Voahangy Rasolofo, Niaina Rakotosamimanana

**Affiliations:** 1 Mycobacteriology Unit, Institut Pasteur de Madagascar, Ambatofotsikely, 101 Antananarivo, Madagascar; 2 Swiss Tropical and Public Health Institute, P.O. Box, 4051 Basel, Switzerland; 3 University of Basel, P.O. Box, 4003 Basel, Switzerland; 4 Immunopathology Axis, Centre de Recherche du Centre Hospitalier de l’Université de Montréal, 900 rue Saint-Denis, Montréal, H2X 3H8 Canada; 5 Microbiology, Infectious Diseases and Immunology Department, Université de Montréal, 2900 Boulevard Edouard Montpetit, Montreal, H3T 1J4 Canada; 6 Madagascar National Tuberculosis Control Programme, Analakely, 101 Antananarivo, Madagascar; 7 Epidemiology Unit, Institut Pasteur de Madagascar, Ambatofotsikely, 101 Antananarivo, Madagascar

**Keywords:** Cascade of care, Drug susceptibility testing, Epidemiology, Madagascar, Multidrug-resistant tuberculosis, Surveillance

## Abstract

**Background:**

In Madagascar, the multidrug-resistant tuberculosis (MDR-TB) surveillance programme was launched in late 2012 wherein previously treated TB cases and symptomatic MDR-TB contacts (hereafter called presumptive MDR-TB cases) undergo drug susceptibility testing. This retrospective review had per aim to provide an update on the national MDR-TB epidemiology, assess and enhance programmatic performance and assess Madagascar’s MDR-TB cascade of care.

**Methods:**

For 2012–2017, national TB control programme notification, clinical management data and reference laboratory data were gathered. The development and coverage of the surveillance programme, the MDR-TB epidemiology and programmatic performance indicators were assessed using descriptive, logistic and spatial statistical analyses. Data for 2017 was further used to map Madagascar’s TB and MDR-TB cascade of care.

**Results:**

The geographical coverage and diagnostic and referral capacities of the MDR-TB surveillance programme were gradually expanded whereas regional variations persist with regard to coverage, referral rates and sample referral delays. Overall, the rate of MDR-TB among presumptive MDR-TB cases remained relatively stable, ranging between 3.9% in 2013 and 4.4% in 2017. Most MDR-TB patients were lost in the second gap of the cascade pertaining to MDR-TB cases reaching diagnostic centres but failing to be accurately diagnosed (59.0%). This poor success in diagnosis of MDR-TB is due to both the current use of low-sensitivity smear microscopy as a first-line diagnostic assay for TB and the limited access to any form of drug susceptibility testing. Presumptive MDR-TB patients’ sample referral took a mean delay of 28 days before testing. Seventy-five percent of diagnosed MDR-TB patients were appropriately initiated on treatment, and 33% reached long-term recurrence-free survival.

**Conclusions:**

An expansion of the coverage and strengthening of MDR-TB diagnostic and management capacities are indicated across all regions of Madagascar. With current limitations, the surveillance programme data is likely to underestimate the true MDR-TB burden in the country and an updated national MDR-TB prevalence survey is warranted. In absence of multiple drivers of an MDR-TB epidemic, including high MDR-TB rates, high HIV infection rates and inter-country migration, Madagascar is in a favourable starting position for MDR-TB control and elimination.

## Background

Madagascar exemplifies some of the main challenges in tuberculosis (TB) and multidrug-resistant TB (MDR-TB) surveillance. Nationally representative data on MDR-TB date back to the first and only national drug resistance survey conducted in 2005–2006. At that time, the MDR-TB rate was 0.2% among new cases (i.e. primary resistance) and 3.4% among previously treated cases (including secondary resistance) [1]. In 2018, according to World Health Organization (WHO) estimates, the combined rates of MDR-TB and rifampicin-resistant TB had reached 0.5% for primary and 5.9% for secondary resistance, leading to a total incidence of 1.6/100,000 population [2]. Beyond these estimates, which rely on sparse quantity and quality of data, the understanding of the drug-resistant TB epidemiology in Madagascar relies on (i) a one-time surveillance data review with limited scope from 2014, (ii) small-scale studies focusing on urban subpopulations and (iii) nationwide limited capacity for routine drug susceptibility testing (DST), including rifampicin resistance testing with GeneXpert MTB/RIF (Cepheid, CA, USA) [3, 4]. Therefore, surveillance of TB drug resistance in Madagascar is lacking behind standards put forward by the WHO [5].

In September 2012, the Madagascar national TB control programme (NTP) launched the MDR-TB surveillance programme [4]. Within this programme, clinical samples of presumptive MDR-TB patients—defined as previously treated cases and symptomatic MDR-TB contacts—undergo DST in regional or national reference laboratories. The real-time surveillance of drug resistance and the timely analysis of the wealth of paper-based data on a quarterly basis can be a challenge for an NTP that is focusing predominantly on service delivery, such as in Madagascar. Yet, periodic review of national drug resistance data can be used to assess and enhance programmatic performance. Tools such as geographic information systems (GIS) that combine epidemiological, infrastructural and geospatial data enable rapid visualisation and understanding of disease epidemiology and programme development in terms of coverage and referral rates [6, 7]. The cascade of care approach has become another useful tool helping countries to comprehensively exploit national data and visualise deficiencies at every step of a patients’ pathway to cure [8]. In India and South Africa, this approach has identified and quantified gaps in TB case detection, case holding and disease outcome and thus supported the formulation of priority interventions [9, 10].

Here, we used NTP data from 2012 to 2017 and applied GIS and cascade of care approaches to (i) describe the infrastructures and processes of Madagascar’s MDR-TB surveillance programme, (ii) assess its deployment in space and time, (iii) measure key laboratory pre- and post-analytic performance indicators, (iv) provide an update on the national MDR-TB epidemiology and (v) assess Madagascar’s TB and MDR-TB cascade of care for 2017.

## Methods

### Ethical considerations

Data was collected as part of Madagascar’s NTP routine health information system under national public health law. Data was anonymised and aggregated precluding identification of patients at individual level. No patient informed consent or ethical review board evaluation was warranted.

### Study area

In Madagascar, with its 26 million inhabitants, some favourable conditions for TB control prevail, including its insular status, a low HIV prevalence and a low MDR-TB prevalence [2, 11, 12]. However, the country is characterised by several conditions favourable for ongoing TB transmission, including (i) a weak health system, (ii) a large proportion of the population living in rural and remote areas with difficult access to healthcare, (iii) poverty and associated factors such as overcrowding, (iv) paucity of human resource capacities with training in TB care, (v) low coverage of TB diagnostic and treatment facilities, (vi) suboptimal observation of treatment and follow-up and (vii) high prevalence of important TB risk factors, such as undernutrition and indoor air pollution [13–15].

### Case definitions and testing algorithms

According to Malagasy NTP guidelines, the WHO reporting framework for TB and in agreement with WHO definitions, case definitions are as follows:

- A confirmed TB case is either a bacteriologically confirmed (smear microscopy, GeneXpert MTB/RIF, culture) or clinically diagnosed case.
- A presumptive MDR-TB case is defined as (i) a previously treated TB case (i.e. disease relapse, treatment failure and retreatment patients according to WHO definitions) or (ii) a symptomatic MDR-TB contact [16].
- A confirmed MDR-TB case is a bacteriologically confirmed TB case with laboratory confirmation of drug resistance to rifampicin and isoniazid [16]. In case of discrepancies between DST methods, culture-based DST is used as the gold standard.

In Madagascar, within the MDR-TB surveillance programme, only clinical samples of presumptive MDR-TB patients undergo DST. Two different diagnostic algorithms were sequentially implemented during the study period (Additional file 1: Fig. S1 and Additional file 2: Fig. S2) [4, 17, 18]. Based on these algorithms, samples submitted for reference testing within the programme between 2012 and 2017 were tested on phenotype DST for first-line drugs, on GeneXpert MTB/RIF for rifampicin resistance and on a combination of phenotype and line probe assay (HAIN LifeSciences, Nehren, Germany) for second-line drugs. As a consequence of the slightly differing algorithms, second-line phenotypic and Genotype MTBDR™ line probe assay results were not available for all included samples.

### Data collection

Data was collected for the period between September 2012 and December 2017. First, the NTP organisation, infrastructures, development of the geographical coverage over time and the overall implementation, including major events that could have influenced programmatic performance, were documented with the NTP staff and personnel of the national reference laboratory through interviews. Second, aggregated case notification and clinical management data of all TB diagnostic and treatment centres across Madagascar were obtained from the NTP in digital form. This included total number of reported TB cases as well as presumptive MDR-TB cases. Third, diagnosis and DST data were obtained from Madagascar’s national reference laboratory for mycobacteria (NRLM). Situated in the capital city of Antananarivo and hosted by the Institut Pasteur de Madagascar, this is the country’s only laboratory with DST capacity beyond GeneXpert MTB/RIF testing. Data included patients’ age and gender, TB clinical presentation, indication for DST, geographic location of the referring diagnostic and treatment centre, Löwenstein-Jensen and MGIT™ (Beckton Dickenson, Franklin Lakes, NJ, USA) phenotyping, GeneXpert MTB/RIF and first- and second-line Genotype MTBDR™ line probe assay results. Finally, the NTP MDR-TB clinical management data provided patient data on treatment regimen, treatment duration, per treatment microbiologic control testing, treatment outcome and HIV status.

### Data analysis

Quantitative (e.g. NTP notification data) and qualitative (e.g. interviews, algorithms) data were combined to describe the nature and reconstruct the historic development of the MDR-TB surveillance programme. A GIS was built with ArcGIS version 10.4.1 (ESRI; Meudon Cedex, France) using the geographical database of the Madagascar Ministry of Public Health and administrative boundaries of the regions available through the Office for the Coordination of Humanitarian Affairs website. Location of MDR-TB surveillance programme infrastructures, including year of implementation, and multidrug-resistance surveillance data by year and region of origin of patients were integrated into the GIS to map the development of the implementation of the MDR-TB surveillance programme.

Sample referral, transportation and testing delays between peripheral diagnosis and treatment centres and the NRLM were compared between regions using descriptive statistics, including means, standard deviations (SDs) and 95% confidence intervals (CIs). A univariable regression model was built using previous treatment history as primary predictor to identify risk of MDR-TB acquisition among the different groups, without adjusting for other explanatory variables.

Demographic and clinical data of all laboratory-confirmed MDR-TB samples were aggregated. Appropriate referral of sputum samples for per-treatment microbiologic follow-up testing among confirmed MDR-TB cases was assessed by confronting MDR-TB patients’ data on TB diagnosis and DST from the NRLM to the NTP MDR-TB clinical management data.

Supranational WHO data, NTP notification and clinical management data, NRLM data on TB diagnosis and DST, previously published and locally assessed laboratory assays’ performance characteristics and systematic review data on the natural history of TB disease were used to map Madagascar’s TB and MDR-TB cascade of care for 2017 only [19, 20]. As recently proposed, the number of patients with prevalent active disease, reaching and being evaluated at TB diagnostic facilities, being diagnosed with disease, accessing treatment, completing treatment and achieving recurrence-free survival were used as the specific ‘steps’ of the cascade for TB and MDR-TB care [21]. Cascade steps, used data sources and corresponding estimates are presented in Table 1. In case of discrepancy between the different data sources, investigators agreed on most reliable data sources to construct the cascade. All calculations, discrepancies and limitations are described in detail in Additional file 3: Appendix S3. CIs around proportions were calculated using the modified Wald method.

**Table 1:** MDR-TB cascade of care data sources and estimations, Madagascar, 2017

Cascade step	Methods/data source	References	TB all forms	Presumptive MDR-TB	MDR-TB
			***n*** (95% CI)	***n*** (95% CI)	***n*** (95% CI)
1. Prevalent cases	• WHO • National drug resistance survey • National reference laboratory data • NTP case notification data	[1, 2, 22, 23]	61,880 (40,040–88,400)	3646 (2258–5437)	341 (61–913)
2. Reached diagnostic and treatment centre	• Country tuberculosis guidelines • Diagnostic literature • Locally assessed sensitivity of sputum microscopy and GeneXpert MTB/RIF • National reference laboratory data • NTP case notification data	[17, 19, 20, 23–25]	51,419 (46,744–57,250)	3563 (3197–4046)	224 (170–330)
3. Diagnosed	• National reference laboratory data • NTP case notification data	[23, 26]	35,339 (35,124–35,570)	2485 (2443–2540)	24 (22–27)
4. Registered for treatment	• NTP clinical management data	[26]	32,427 (−)	2257 (−)	18 (−)
5. Cured or treatment completed	• NTP clinical management data	[26]	28,178 (−)	1852 (−)	8 (−)
6. Recurrence-free survival	• NTP clinical management data • Clinical TB literature	[26, 27]	27,996 (27,774–28,108)	1670 (1448–1782)	8 (−)

The cascade of care steps with references and total number of cases. Presumptive MDR-TB cases are included in TB all forms; MDR-TB cases are included in presumptive MDR-TB cases

*CI* confidence interval, *MDR-TB* multidrug-resistant tuberculosis, *MTB/RIF Mycobacterium tuberculosis*/rifampicin, *NTP* national tuberculosis control programme, *WHO* World Health Organization

All statistical analyses were performed using Stata version 14.0 (Stata Corporation, College Station, USA).

## Results

### Nature and development of the MDR-TB surveillance programme

**Fig. 1:**
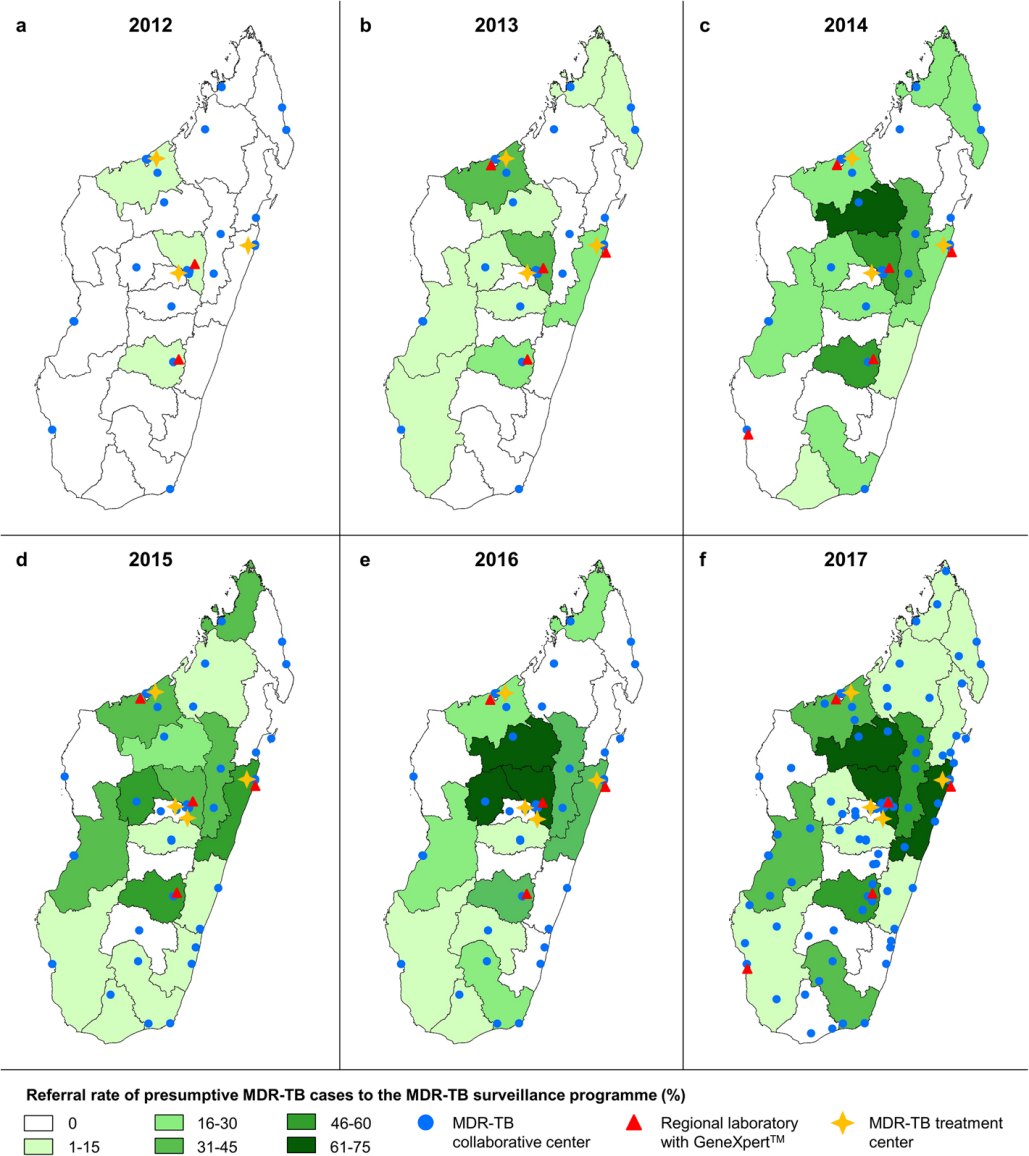
Development of MDR-TB surveillance programme infrastructures and referral rates of presumptive MDR-TB patients, MDR-TB surveillance programme, Madagascar, 2012–2017. **a** 2012. **b** 2013. **c** 2014. **d** 2015. **e** 2016. **f** 2017

In Madagascar, symptomatic TB patients initially self-present to a diagnostic and treatment centre (intermediate level facility) which can diagnose TB based on smear microscopy and can implement directly observed therapy for cases of drug-susceptible TB [18, 28]. In 2017, there were 219 TB diagnostic and treatment centres across Madagascar (compared to 215 in 2012), translating into 0.8 centres per 100,000 habitants (Fig. 1).

From those diagnostic and treatment centres, presumptive MDR-TB patients are referred to MDR-TB collaborative centres, i.e. referral centres for potentially drug-resistant TB cases. Collaborative centres possess identical laboratory infrastructure as diagnostic and treatment centres but are accredited to collect and send sputum samples to the nearest out of seven regional reference laboratories with GeneXpert MTB/RIF testing capacity. Sputum samples from presumptive MDR-TB cases are referred to the MDR-TB surveillance programme if positive on smear microscopy or if highly suspected clinically. At programme onset, 25 (11%) diagnostic and treatment centres were selected and trained to act as MDR-TB collaborative centres. The number of collaborative centres was rapidly increased to 37 in 2013, and as of 2017, 101 MDR-TB collaborative centres existed, corresponding to 46% of all TB diagnostic and treatment centres in the country [4].

The regional reference laboratories send the sputum further to the NRLM for culture and phenotypic first- and second-line DST, regardless of the initial molecular testing results. In case of MDR-TB confirmation, the NRLM communicates the result to the NTP, which in turn informs the referring diagnostic and treatment centre. That centre is then responsible for transferring the patients to one of the country’s four MDR-TB treatment centres. MDR-TB patients are usually hospitalised during the 6-month intensive phase of their treatment, and thereafter, they receive 12 months directly observed treatment at their closest diagnostic and treatment centre.

Figure 1a–f present the distribution and development of MDR-TB collaborative centres, regional reference laboratories equipped with a GeneXpert MTB/RIF and treatment centres between 2012 and 2017. The processes within the MDR-TB surveillance programme are presented as Additional file 4: Fig. S4.

### MDR-TB surveillance programme uptake

**Fig. 2:**
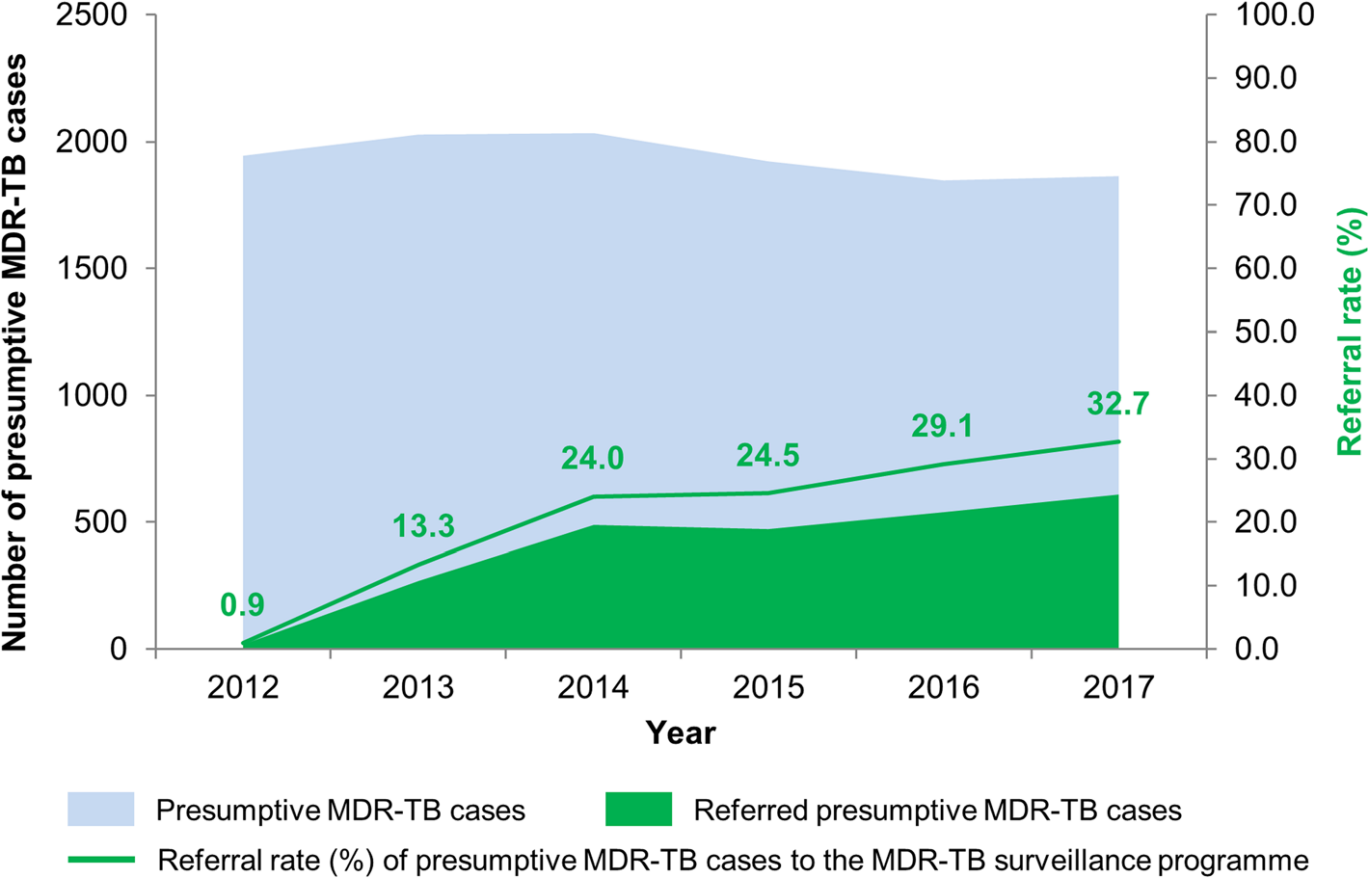
Absolute number of indicated and referred presumptive MDR-TB cases and associated referral rate, MDR-TB surveillance programme, Madagascar, 2012–2017

According to NTP case notification data, referral to the MDR-TB surveillance programme was indicated for 11,643 TB patients (i.e. presumptive MDR-TB cases) between 2012 and 2017. Thereof, an overall of 2391 patients (20.5%) were factually referred for MDR-TB evaluation whilst an evaluation was missed for the remaining 79.5%. Absolute number of referrals to the MDR-TB surveillance programme increased steadily from 20 in 2012 to 269 in 2013 and to 610 in 2017 (Fig. 2). Accordingly, the referral rate evolved from 13.3% in 2013 to 24.0% in 2014, to 24.5% in 2015, to 29.1% in 2016 and to 32.7% in 2017. Regional differences in referral rates of presumptive MDR-TB patients by year are displayed in Fig. 1. For some regions, no samples were referred for DST during the study period despite implementation of collaborative centres.

Table 2 describes the demographic, clinical and laboratory information for patients enrolled in the surveillance programme (i.e. presumptive MDR-TB cases). The majority of included patients were relapse cases (61.7%), followed by failure (13.9%) and retreatment cases (10.2%). The majority of samples taken were for suspicion of pulmonary TB (96.7%).

**Table 2:** Characteristics and resistance profiles of presumptive MDR-TB patients, MDR-TB surveillance programme, Madagascar, 2012–2017

	2012	2013	2014	2015	2016	2017	Total
	***n*** (%)	***n*** (%)	***n*** (%)	***n*** (%)	***n*** (%)	***n*** (%)	***n*** (%)
**Total**	**20**	**268**	**488**	**469**	**536**	**610**	**2391**
***Gender***							
Male	12 (60.0)	195 (73.3)	326 (66.9)	302 (65.4)	359 (67.0)	426 (70.0)	1620 (68.1)
Female	8 (40.0)	71 (26.7)	161 (33.1)	160 (34.6)	177 (33.0)	183 (30.0)	760 (31.9)
***Median age (in years ± SD)***	45 ± 16.4	40 ± 13.2	40 ± 13.3	39 ± 14.4	40 ± 14.2	41 ± 14.7	40 ± 14.1
***Previous treatment history***							
Relapse	4 (20.0)	107 (39.9)	257 (52.7)	302 (64.4)	376 (70.2)	430 (70.5)	1476 (61.7)
Failure	8 (40.0)	69 (25.8)	82 (16.8)	58 (12.4)	43 (8.0)	73 (12.0)	333 (13.9)
Retreatment	0 (0.0)	21 (7.8)	53 (10.9)	54 (11.5)	54 (10.1)	61 (10.0)	243 (10.2)
Unknown	8 (40.0)	71 (26.5)	96 (19.7)	55 (11.7)	63 (11.8)	46 (7.5)	339 (14.2)
***Clinical form***							
Pulmonary	20 (100.0)	249 (93.6)	482 (98.8)	452 (96.6)	526 (98.1)	580 (95.2)	2309 (96.7)
Extrapulmonary	0 (0.0)	17 (6.4)	6 (1.2)	16 (3.4)	10 (1.9)	29 (4.8)	78 (3.3)
***MTBC identification***							
MTBC	16 (84.2)	229 (85.5)	440 (90.2)	367 (78.6)	438 (81.7)	478 (78.4)	1968 (82.4)
Non-MTBC	3 (15.8)	39 (14.5)	48 (9.8)	100 (21.4)	98 (18.3)	132 (21.6)	420 (17.6)
***Drug resistance profiles***							
Pan-susceptible	9 (56.3)	199 (86.9)	392 (89.1)	331 (90.2)	368 (84.0)	427 (89.3)	1726 (87.7)
Isoniazid monoresistance	3 (18.8)	11 (4.8)	24 (5.5)	14 (3.8)	38 (8.7)	23 (4.8)	113 (5.7)
Rifampicin monoresistance	0 (0.0)	3 (1.3)	6 (1.4)	4 (1.1)	7 (1.6)	6 (1.3)	26 (1.3)
MDR-TB	3 (18.8)	13 (5.7)	17 (3.9)	16 (4.4)	19 (4.3)	21 (4.4)	89 (4.5)
Pre-XDR-TB	0 (0.0)	1 (0.4)	1 (0.2)	1 (0.3)	0 (0.0)	0 (0.0)	3 (0.2)
XDR-TB	0 (0.0)	0 (0.0)	0 (0.0)	0 (0.0)	0 (0.0)	0 (0.0)	0 (0.0)
Other^1^	1 (0.1)	2 (0.1)	0 (0.0)	1 (0.1)	6 (0.3)	1 (0.1)	11 (0.5)

Data missing for gender (*n* = 11), clinical form (*n* = 4) and MTBC identification (*n* = 3)

*MTBC Mycobacterium tuberculosis* complex, *MDR-TB* multidrug-resistant tuberculosis, *XDR-TB* extensive drug-resistant tuberculosis, *SD* standard deviation

^1^Resistances to other drugs tested: aminoglycosides, ethambutol, ofloxacin, streptomycin

### Sample referral delays

**Fig. 3:**
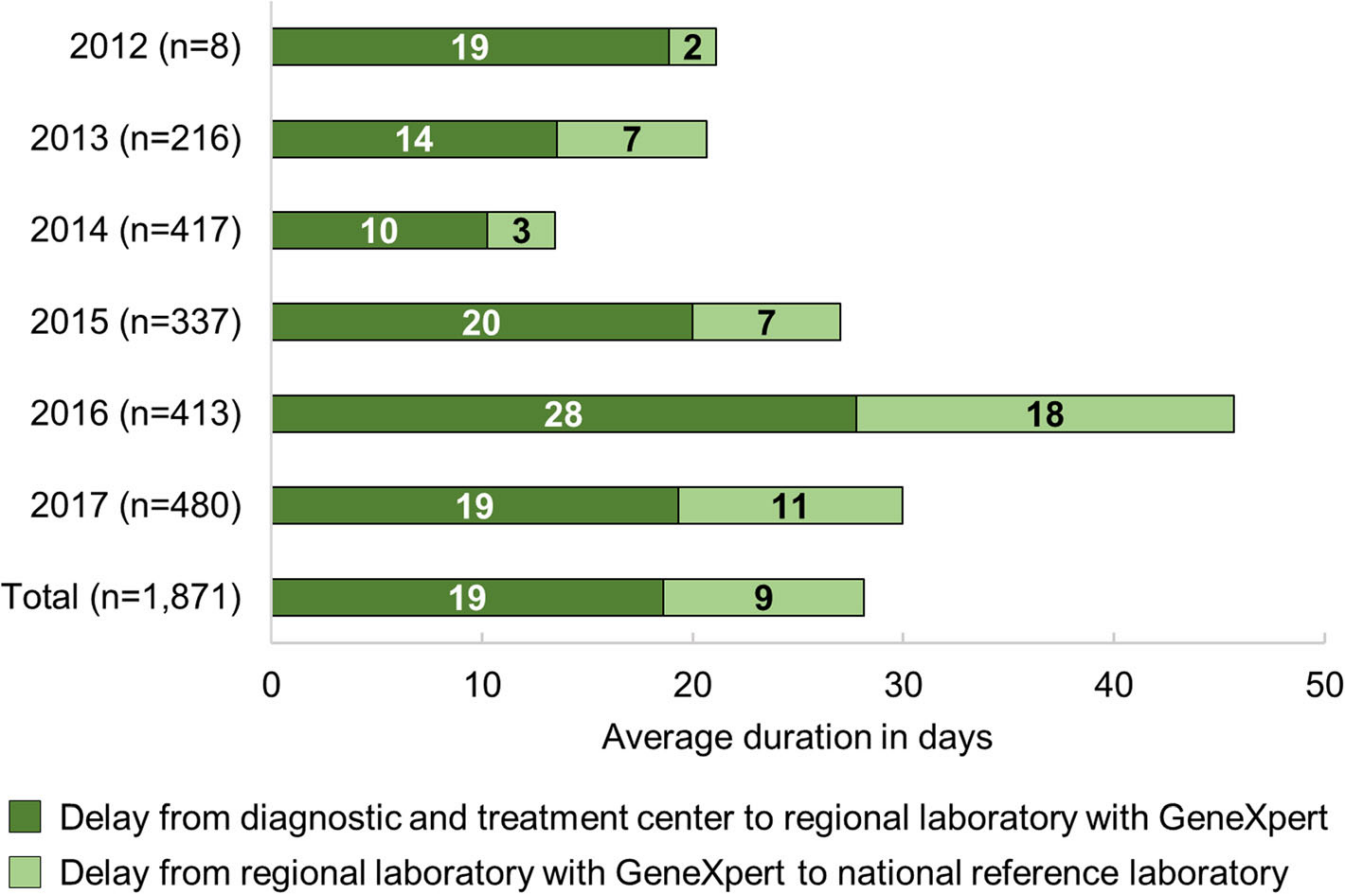
Sample referral delays, MDR-TB surveillance programme, Madagascar, 2012–2017

Sample referral delays are divided between (i) from a diagnostic and treatment centre to a GeneXpert MTB/RIF regional laboratory and (ii) from a GeneXpert MTB/RIF regional laboratory to the NRLM (Fig. 3). All years combined, it took samples a mean of 19 days (± 25 SD) to reach a GeneXpert MTB/RIF regional laboratory and another 9 days (± 15 SD) to reach the NRLM. Even in Analamanga, the capital region, where the NRLM is located, the mean sample referral delay was 15 days (± 16 SD) between 2012 and 2017 and 20 days (± 16 SD) in 2017 alone.

### Monoresistant TB, MDR-TB and XDR-TB

**Fig. 4:**
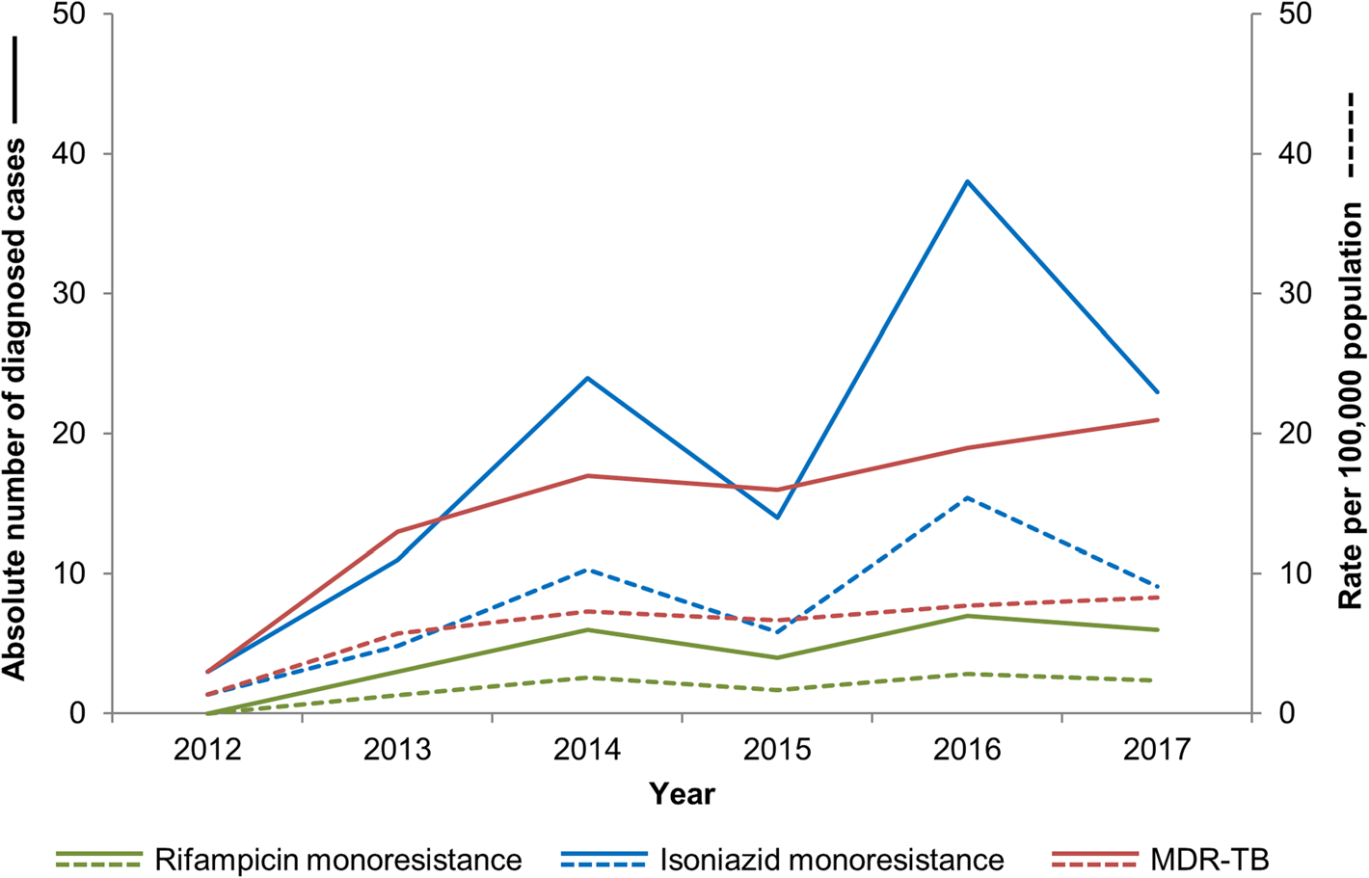
Diagnosed rifampicin monoresistance, isoniazid monoresistance and multidrug-resistance, MDR-TB surveillance programme, Madagascar, 2012–2017

Between 2012 and 2017, 2391 samples were received at the NRLM and bacteria from the TB complex were confirmed by culture, GeneXpert MTB/RIF and/or HAIN assays in 1968 (82.4%) of those (Table 2). DST found 87.7% (1726/1968) of all isolates to be pan-susceptible and 7.0% (139/1968) were resistant to one first-line drug. Thereof, 5.7% were resistant to isoniazid and 1.3% to rifampicin. No monoresistance to ethambutol was detected whilst DST for pyrazinamid was not performed. Isoniazid monoresistance rates fluctuated strongly between years, but there was an overall increasing trend from 4.8 to 9.1 per 100,000 population between 2013 and 2017, as depicted in Fig. 4. The increasing trend was less accentuated for rifampicin monoresistance. Over 2012–2017, 89 (4.5%) isolates were MDR-TB and trends showed a relatively stable rate over the observed period. Three cases (0.2%) were found to present a pre-XDR-TB profile, namely being resistant to isoniazid *and* rifampicin *and* fluoroquinolones (*n* = 3) or injectable aminoglycosides (*n* = 0). XDR-TB was not yet diagnosed in Madagascar.

Table 3 describes MDR-TB patient characteristics. The majority of MDR-TB patients were males (67.4%; 60/89) whereas the MDR-TB rate was similar in men and women (3.7% and 3.8%, respectively). Overall, 2.6% of relapse, 9.9% of failure and 1.6% of retreatment cases for which a sample was submitted to the MDR-TB surveillance programme were confirmed to be MDR-TB. Hence, failure cases were at significant higher risk to have MDR-TB as compared to relapse cases as they had 3.87 times higher odds of confirmed MDR-TB (95% CI 2.39–6.28).

**Table 3:** MDR-TB patient characteristics, MDR-TB surveillance programme, Madagascar, 2012–2017

	Presumptive MDR-TB	MDR-TB		Univariable logistic regression	
	***n*** (%)	***n***	% (95% CI)	Unadjusted OR (95% CI)	***p***-value
**Total**	**2391 (100.0)**	**89**	**3.7 (3.0–4.6)**	**–**	**–**
***Gender***					
Male	1620 (68.1)	60	3.7 (2.8–4.7)	1.00 (reference population)	
Female	760 (31.9)	29	3.8 (2.6–5.4)	1.06 (0.67–1.67)	0.802
***Median age (in years [range])***	40 [2–88]	40 [12–67]	–	–	–
***Previous treatment history***					
Relapse	1476 (61.7)	39	2.6 (1.9–3.6)	1.00 (reference population)	
Failure	333 (13.9)	33	9.9 (6.9–13.6)	3.87 (2.39–6.28)	< 0.001
Retreatment	243 (10.2)	4	1.6 (0.5–4.2)	0.58 (0.21–1.64)	0.304
Unknown	339 (14.2)	13	3.8 (2.1–6.5)	1.70 (0.90–3.24)	0.104

*CI* confidence interval, *MDR-TB* multidrug-resistant tuberculosis, *OR* odds ratio

**Fig. 5:**
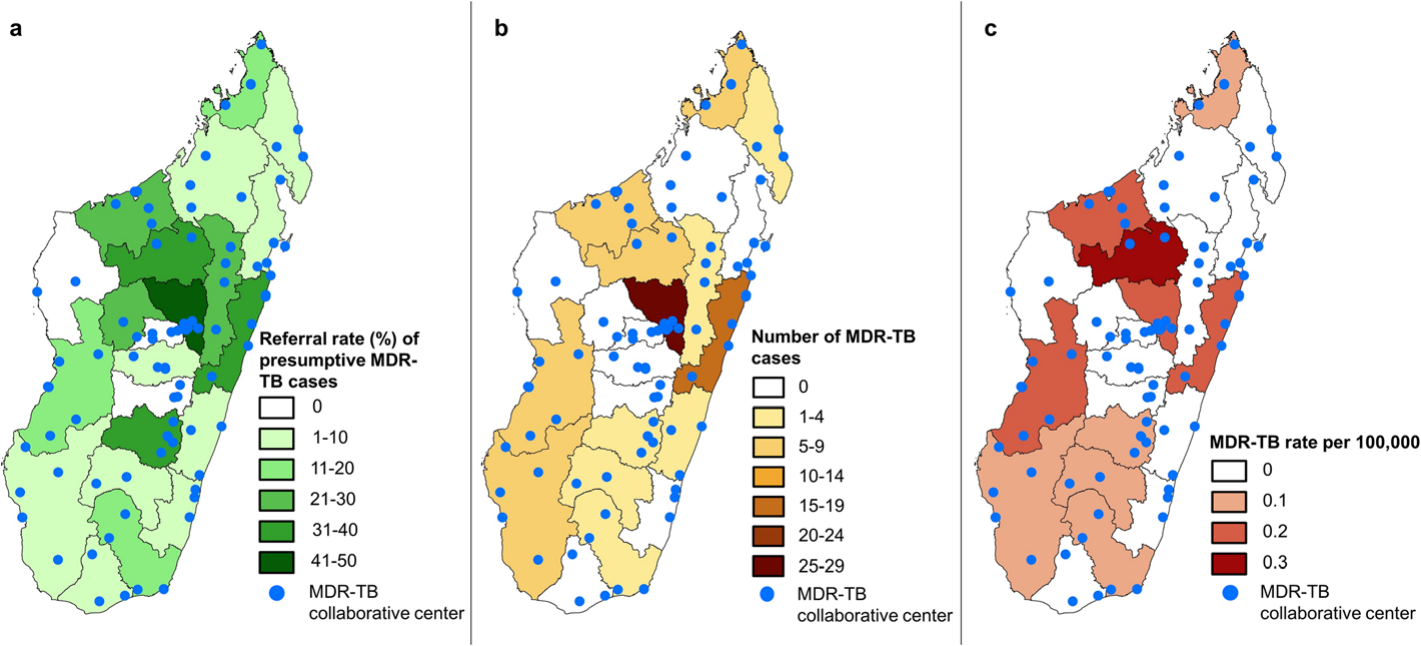
Geographic distribution of MDR-TB cases per region, MDR-TB surveillance programme, Madagascar, 2012–2017. **a** Referral rates of presumptive MDR-TB cases. **b** MDR-TB patients’ absolute numbers. **c** MDR-TB incidence rate per 100,000 population

Figure 5a–c display how increased referral and testing rates translated into increased MDR-TB diagnosis. Figure 5a shows the pooled referral rates for 2012–2017 for each region. Figure 5b shows the cumulative absolute number of MDR-TB cases diagnosed in Madagascar 2012–2017 by region, and Fig. 5c shows the corresponding MDR-TB incidence rates per 100,000 population by region over the same time period.

### Cascade of care

**Fig. 6:**
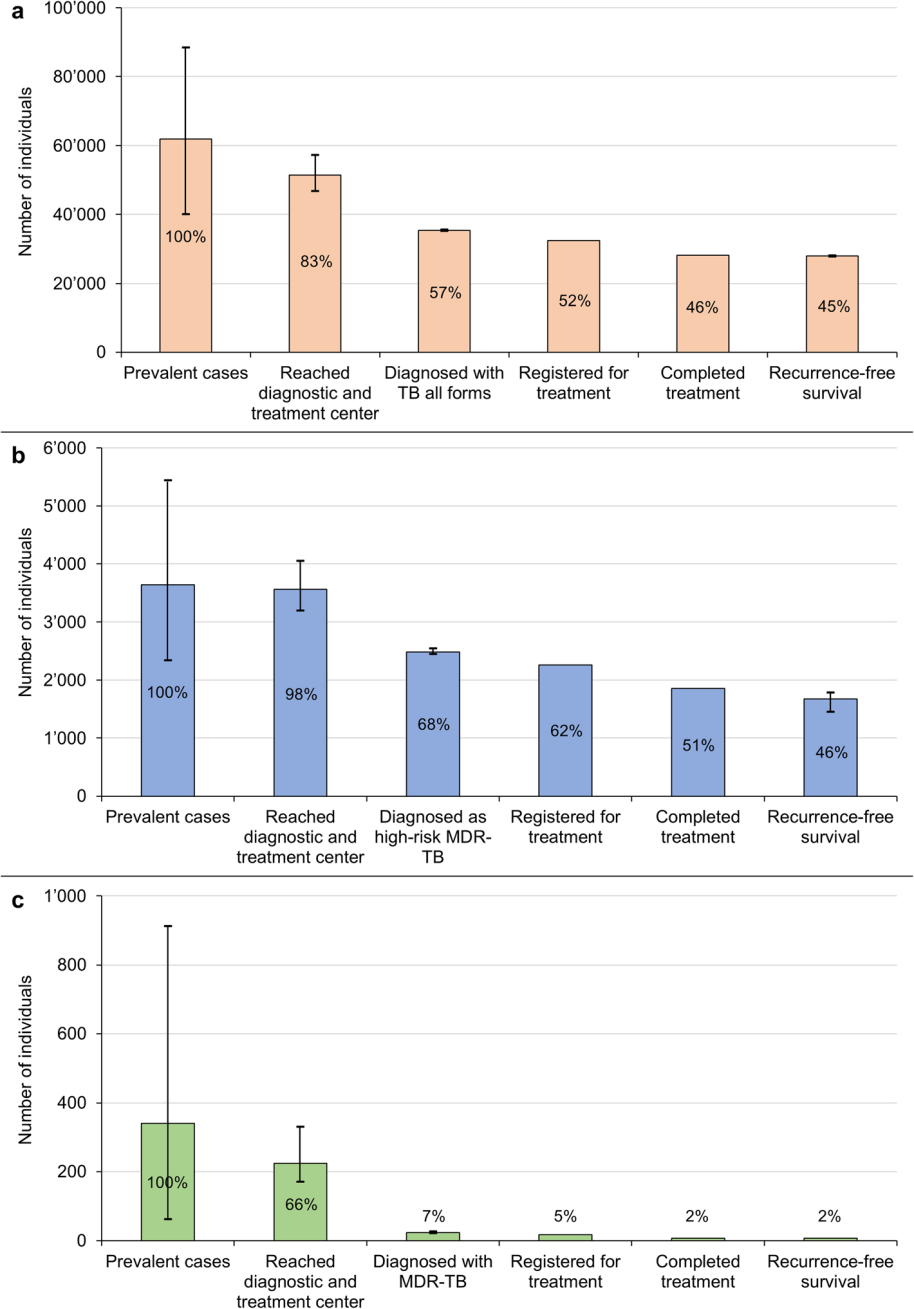
Cascade of care, Madagascar, 2017. **a** TB all forms. **b** Presumptive MDR-TB cases. **c** MDR-TB patients. Percentage represent the amount of patients lost at every gap of the cascade of care (e.g. 17% of prevalent cases of TB all forms do not reach diagnostic and treatment centres and 26% of patients reaching diagnostic and treatment centres are not diagnosed)

Madagascar’s cascade of care was constructed using a standardised model for the 2017 period [21]. Table 1 presents the used data sources, references and corresponding number of cases at every step of the cascade. Three distinct cascades were build: TB all forms (Fig. 6a), presumptive MDR-TB cases (Fig. 6b) and confirmed MDR-TB patients (Fig. 6c).

#### TB cases of all forms

In the absence of a national TB prevalence study, WHO estimates that there were 61,880 (95% CI 40,040–88,400) prevalent TB cases in the country in 2017 (Fig. 6a). Surrogate markers of TB prevalence such as centralised tracking of anti-TB drug distribution or universal healthcare digital system data were not available to corroborate the WHO and NTP data in Madagascar. NTP case notification data, NTP diagnosis algorithms and NRLM diagnosis data were used to estimate the number of smear-positive patients being evaluated at diagnostic and treatment centres. Smear-positive to smear-negative ratios in the diagnosed population were used to infer the number of smear-negative patients being evaluated at diagnostic and treatment centres. We estimate that 51,419 (95% CI 46,744–57,250) patients with active TB disease accessed diagnostic and treatment centres. This represents an estimated 10,461 (16.9%) of TB patients going missing without any interaction with the health system. A total of 35,339 (95% CI 35,124–35,570) were appropriately diagnosed with TB. This was estimated using Madagascar NTP notification data and calculated average loss to follow-up rates, assuming those rates are constant following diagnosis and until treatment completion. As per Madagascar NTP clinical management data, 32,427 and 28,178 patients were, respectively, registered for treatment and appropriately followed until treatment completion. Madagascar’s NTP does not collect post-treatment, long-term follow-up data for TB patients. The country’s overall retreatment rates and literature data on the rates of early versus late relapse were used to estimate that 27,996 (95% CI 27,774–28,108) patients had successfully progressed towards 12–24 months recurrence-free survival.

#### Presumptive MDR-TB cases

Using the ratio of presumptive MDR-TB patients notified by the NTP and the total WHO number of prevalent cases, we estimate there were 3646 (95% CI 2258–5437) cases of presumptive MDR-TB in Madagascar in 2017. Using the same estimation as for TB all forms, 3563 (95% CI 3197–4046) of those reached a TB diagnosis centre and 2485 (95% CI 2443–2540) were diagnosed with TB and recognised as presumptive MDR-TB patients. Among these presumptive MDR-TB cases, only 610 (24.5%) had a sputum sample sent for testing in the NRLM and were thus appropriately enrolled in the MDR-TB surveillance programme (Tables 1 and 2). According to NTP notification and clinical management data, 2485 presumptive MDR-TB patients were diagnosed, 2257 were appropriately enrolled into treatment and 1852 of those completed therapy. We estimate that 1670 (95% CI 1448–1782) were cured and recurrence-free at 12–24 months.

#### MDR-TB cases

Based on WHO estimates, a previous drug resistance survey, capacity for DST and mono-rifampicin resistance to multidrug-resistance ratios obtained from the NRLM diagnosis and DST data, we estimate that there were 341 (95% CI 61–913) prevalent cases of MDR-TB in Madagascar in 2017. Given the limited inclusion of smear-negative patients in the MDR-TB surveillance programme, we evaluate that 224 (95% CI 170–330) patients reached the diagnosis and treatment centres but only 24 (95% CI 22–27) of them were diagnosed with MDR-TB. According to the 2017 diagnosis algorithm, only culture results were considered for the diagnosis of MDR-TB. Hence, only 18 MDR-TB cases were effectively reported to the NTP and correctly initiated for treatment. Importantly, due to the previous algorithms used, 6 (25.0%) MDR-TB cases were missed and left un- or inappropriately treated. Eight patients (33.3%) completed therapy and were declared as cured. Finally, among treated patients, 6 (33.3%) were lost to follow-up and 4 (22.2%) experienced a fatal outcome, resulting in a mortality rate between 22.2% and 55.5%. No information on long-term recurrence-free survival was available. Hence, we assumed the 8 patients who had completed treatment have survived.

## Discussion

The Madagascar MDR-TB epidemiology and cascade of care were approximated using best available data, NTP documentation and literature. Geospatial and cascade approaches were used to understand the challenges of MDR-TB control in the country and set the ground for targeted control and elimination efforts.

The first gap in the cascade of care model pertaining to prevalent MDR-TB cases that do not reach diagnostic and treatment centres was estimated at 34.3%. In Madagascar, access to healthcare is notoriously limited as up to 60% of the population live in > 5 km distance to a primary healthcare facility and 20% live in enclaved areas [29]. The coverage for TB diagnostic and treatment centres is even more bleak as only 219 of the 2563 primary facilities (8.5%) are diagnosing TB, which can translate to distances of several days of walk to the nearest diagnostic and treatment centre [29, 30]. Since programme onset in 2012, the geographical coverage and diagnostic and referral capacities of the MDR-TB surveillance programme were gradually expanded. This trend is continued beyond the study period, with an additional 67 diagnostic and treatment centres trained to act as collaborative centres until 2019. Notwithstanding significant improvements, there remain large proportions of the population without access to TB and MDR-TB diagnosis and treatment, a major pitfall in disease control [31]. In addition, in contrary to the programme algorithms, only about a fifth of presumptive MDR-TB cases have been referred to the MDR-TB surveillance programme. Furthermore, specific attention is warranted for the referral of extrapulmonary TB cases where very low rates were noted although national data suggests that 21% of TB cases are extrapulmonary. This discrepancy is probably a surrogate marker for the limited available resources of obtaining extrapulmonary clinical samples in diagnostic and treatment centres. In conclusion, both a continued geographical expansion of the MDR-TB surveillance programme coverage and an improvement of its current performance, including staff awareness, training and supervision, are recommended.

The next marked gap in the modelled cascade occurs between the amount of TB patients reaching diagnostic facilities and those being effectively diagnosed with disease where marking losses of 26.0% (all forms of TB) and 58.7% (MDR-TB) are observed. The current use of smear microscopy as a first-line assay and the national underuse of GeneXpert MTB/RIF platforms negatively influence the diagnosis rates of both TB and MDR-TB due to the lower sensitivity of microscopy [20, 32]. The fact that this gap is markedly higher in the MDR-TB cascade reflects the limited referral rates of smear-negative presumptive MDR-TB patients for GeneXpert MTB/RIF and culture reference testing within the MDR-TB surveillance programme. Our GIS approach confirmed that regions and diagnostic and treatment centres that ensure access to DST for more presumptive MDR-TB cases are finding more cases. Consequently, the approach was also able to identify diagnostic and treatment centres that are underperforming in terms of referral to the national MDR-TB surveillance programme.

Universal access to DST is now recommended by WHO, but results show that less than 1% of Malagasy TB patients have access to any form of DST, which is considerably below the global average estimate of 30% [33, 34]. Restricting DST to presumptive MDR-TB patients, as done in Madagascar to date, results in patients with primary resistance cycling through at least one unsuccessful treatment course before their resistance is detected. According to findings of the 2005–2006 national survey, primary resistance was found in 0.2% of TB patients [1]. However, in this study, most MDR-TB cases were treatment failure cases, which could suggest that primary resistance is higher than previously reported.

To date, access to GeneXpert MTB/RIF is limited and requires referral of samples. These referrals are organised by road transport through a service provider or individual porters in areas not served by the service provider. With an average of 30 days for sample referral in 2017, the referral system is exceeding the NTP’s targeted maximum of 5 days by far [18]. These delays might be partly responsible for the high rate (17.6%) of failure to confirm TB on culture on referred samples (Table 2) [1]. Other factors causing this high rate might include (i) false positive smear microscopy testing in the diagnosis and treatment centres, (ii) presence of dead bacilli on initial smear microscopy testing or (iii) culture contamination. However, available data did not allow to test these hypotheses.

The benefits of strengthening referral systems on delays and diagnosis rates have been demonstrated in other low-income countries [35]. In Madagascar, innovative technologies such as drones were recently tested to accelerate access to diagnosis and render TB care more patient-centric by overcoming pertinent logistic barriers on the last mile [36]. In addition, the country was upscaling its GeneXpert MTB/RIF platform capacity from 7 to 15 in 2019 and this expansion is expected to help towards narrowing this gap in the cascade of care.

According to cascade estimates, 75.0% of all diagnosed MDR-TB patients were initiated on treatment. The gap between diagnosis and registration for treatment might be explained by under-reporting, the loss of information between the national TB laboratory and the NTP treatment teams, and the fact that there were only four treatment centres in Madagascar. Thus, patients have to spend the 6 initial months of treatment in isolation potentially far away from their homes, hence representing a barrier to MDR-TB treatment access. However, according to the NTP MDR-TB clinical management data, all MDR-TB cases which were reported to the NTP were initiated on treatment suggesting efficient procedures and communication among the different actors, including the patient.

The mortality rate in treated cases, accounting for unknown outcomes in patients lost to follow-up, ranged between 22.2% and 55.5% in treated cases that is higher than the global average of 15.0% [37].

In Madagascar, the rate of MDR-TB among presumptive MDR-TB cases remained relatively stable over the study period, ranging between 3.9% in 2013 and 4.4% in 2017. The national survey (2005–2006) found a MDR-TB rate in presumptive MDR-TB cases of 3.4% [1]. The cross-sectional nature of the survey is likely to give a more precise picture of the true prevalence of MDR-TB especially considering the suboptimal referral rate of presumptive MDR-TB cases for DST in the surveillance programme. This deficiency is diminishing the internal validity of the surveillance data and potentially underestimates the MDR-TB rate. Accordingly, WHO estimates the MDR-TB rate in this group at 5.9% (2018) [33]. Conclusively, in Madagascar, (i) improved compliance with NTP guidelines and increased referral of presumptive MDR-TB patients for reference DST will likely lead to an increase in MDR-TB cases notifications, (ii) surveillance programme data is likely to underestimate the true MDR-TB burden in the country and (iii) a repetition of the national MDR-TB survey is warranted to update the nationwide MDR-TB situation on primary and secondary resistance.

As of early 2018, Madagascar started to implement the WHO-recommended 9-month ‘short’ treatment regimen for MDR-TB and the here documented low levels of resistance to fluoroquinolones and aminoglycosides bode well for the implementation of this regimen. As new and repurposed drugs, including bedaquiline and linezolid, are now recommended as first-line agents for longer MDR-TB regimens, laboratory capacity for susceptibility testing to those drugs need to be developed in-country [38]. Recent WHO guidelines for second-line drug phenotypic testing and Madagascar’s implementation of TB whole genome sequencing as a reference assay for DST should ensure appropriate development of local capacity for DST to those new and repurposed drugs [39, 40].

In Madagascar, the absence of multiple drivers of the MDR-TB epidemic represents a favourable starting position for control and elimination. First, as described in this study and previous reports including national drug resistance surveys, the national incidence of MDR-TB remains low compared to other low-income country settings [33]. Second, the HIV infection prevalence in Madagascar is low at 0.3% in 15–49-year-olds [12]. Evidence supports an increased risk for TB acquisition, progression to active disease and transmission among HIV-positive individuals as well as unfavourable outcomes among MDR-TB and HIV co-infected patients [12, 41]. Third, the island status of the country excludes proximity to a high-prevalence MDR-TB setting and strongly limits between-country mobility. Thus, no significant introduction of MDR-TB is expected considering the fact that migration is a main driver for infectious diseases [42]. Even though at current stage there are no strong indications that the MDR-TB situation will change rapidly, a potential increase could occur if the status quo for case finding and case holding is maintained. The increasing HIV rates are an alarming trend that could have devastating effects on TB and MDR-TB control [43].

### Limitations

Our study has several limitations. The surveillance data relies on routinely collected data with limited coverage in a pre-defined high-risk group (i.e. presumptive MDR-TB cases); hence, its ability to reflect the true MDR-TB epidemiology in the general population is limited. The cascade of care estimates rely on extensively collected and aggregated data from the country’s NTP for all laboratory, notifications and treatment aspects of the TB programme which led to reliable estimations of the cascade. However, limitations on the calculations and estimations of the different steps and gaps of the cascade of care are described in Additional file 3: Appendix S3. In addition, the reliance of MDR-TB diagnosis on smear microscopy screening has led to significant uncertainty and wide CIs around the number of MDR-TB patients reaching TB diagnostic and treatment centres.

## Conclusions

The findings presented here support the understanding of the MDR-TB epidemiology and cascade of care in Madagascar. The data revealed pitfalls in TB and MDR-TB control that need priority attention, including (i) an expansion of the coverage of diagnostic and management capacities, (ii) improved referral for presumptive MDR-TB cases to the MDR-TB surveillance programme, (iii) reduction of sample referral delays, (iv) improved observation and monitoring of treatment and its outcomes and (v) conduction of a representative national drug resistance survey to elucidate on the true prevalence of MDR-TB in Madagascar [5]. Madagascar and the international TB community are specifically challenged to enable access to state of the art TB diagnostics and DST in conformity with international minimal standards for all Malagasy [44]. Together with programme performance improvements suggested by this study, MDR-TB control in Madagascar is achievable.

## Supplementary information

**Additional file 1.** Fig. S1: 2012–2013 MDR-TB surveillance programme testing algorithm, Madagascar.

**Additional file 2.** Fig. S2: 2014–2017 MDR-TB surveillance programme testing algorithm, Madagascar.

**Additional file 3.** Appendix S3: Calculation of steps and gaps for the TB and MDR-TB cascade of care, Madagascar, 2017.

**Additional file 4.** Fig. S4: Infrastructures and processes, MDR-TB surveillance programme, Madagascar, 2012–2017.

## Data Availability

The datasets used and/or analysed during the current study are available from the corresponding author on reasonable request.

## Abbreviations

DST: Drug susceptibility testing
GIS: Geographical information system
MDR-TB: Multidrug-resistant tuberculosis
NRLM: National reference laboratory for mycobacteria
NTP: National tuberculosis control programme
TB: Tuberculosis
WHO: World Health Organization
XDR-TB: Extensively drug-resistant tuberculosis
