# Supplementary figures and images for: Multidrug-resistant tuberculosis surveillance and cascade of care in Madagascar: a five-year (2012–2017) retrospective study
Source: BMC Med. 2020 Jun 30;18:173. doi: 10.1186/s12916-020-01626-6 (PMC7325144; doi:10.1186/s12916-020-01626-6)

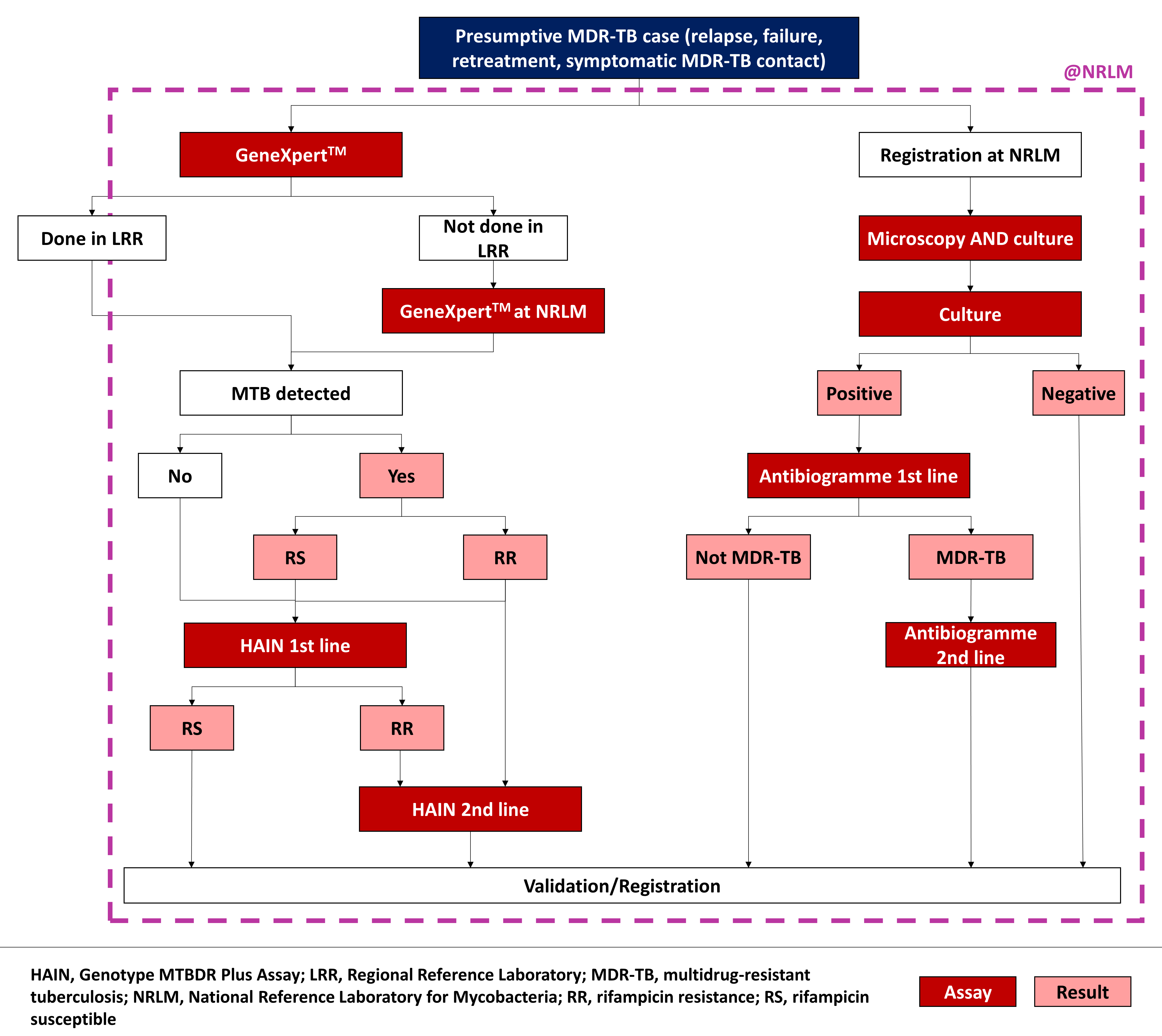

Supplement: Supplementary file 1 — Additional file 1. Fig. S1: 2012–2013 MDR-TB surveillance programme testing algorithm, Madagascar. [file 12916_2020_1626_MOESM1_ESM.tif]

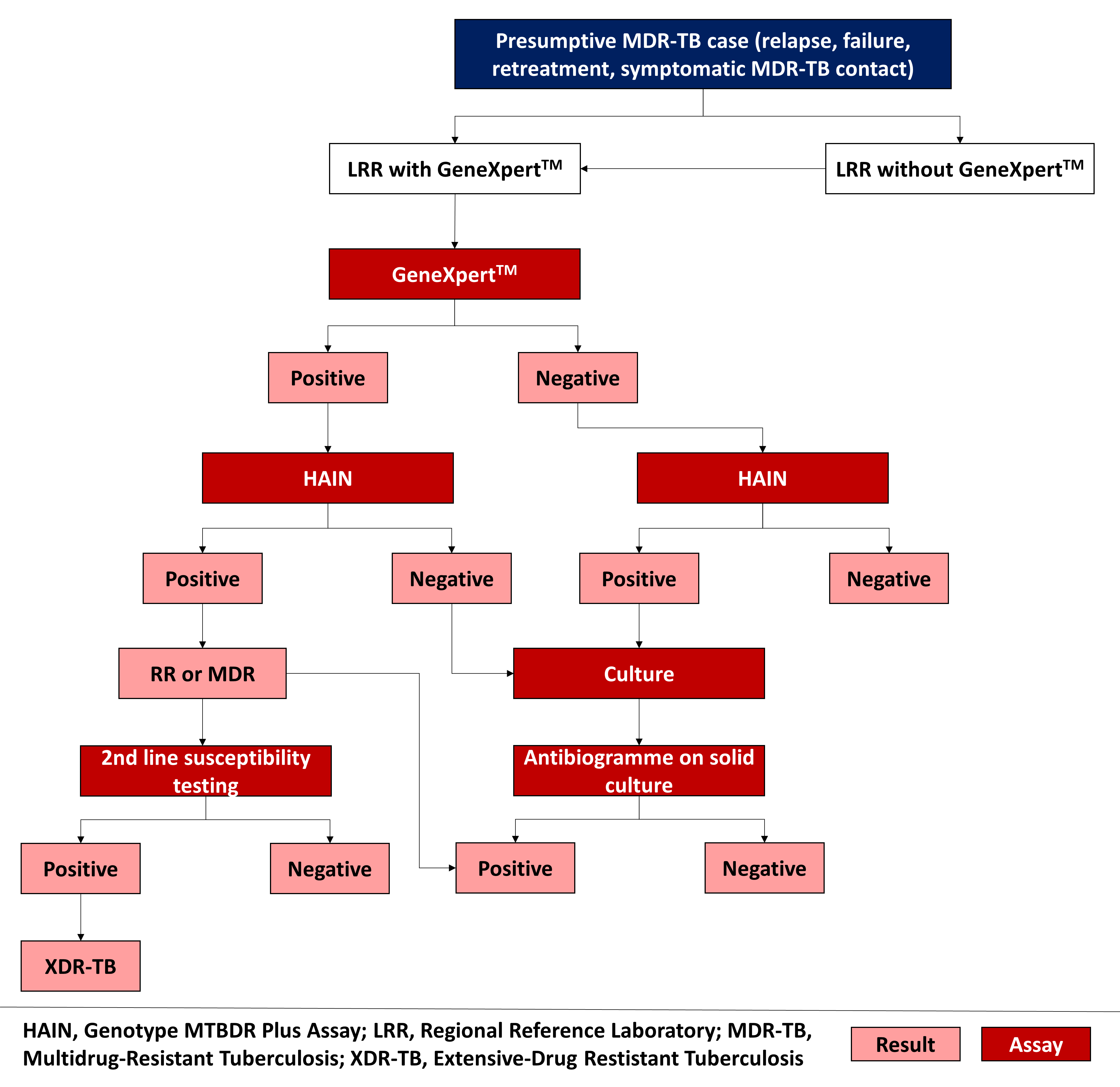

Supplement: Supplementary file 2 — Additional file 2. Fig. S2: 2014–2017 MDR-TB surveillance programme testing algorithm, Madagascar. [file 12916_2020_1626_MOESM2_ESM.tif]

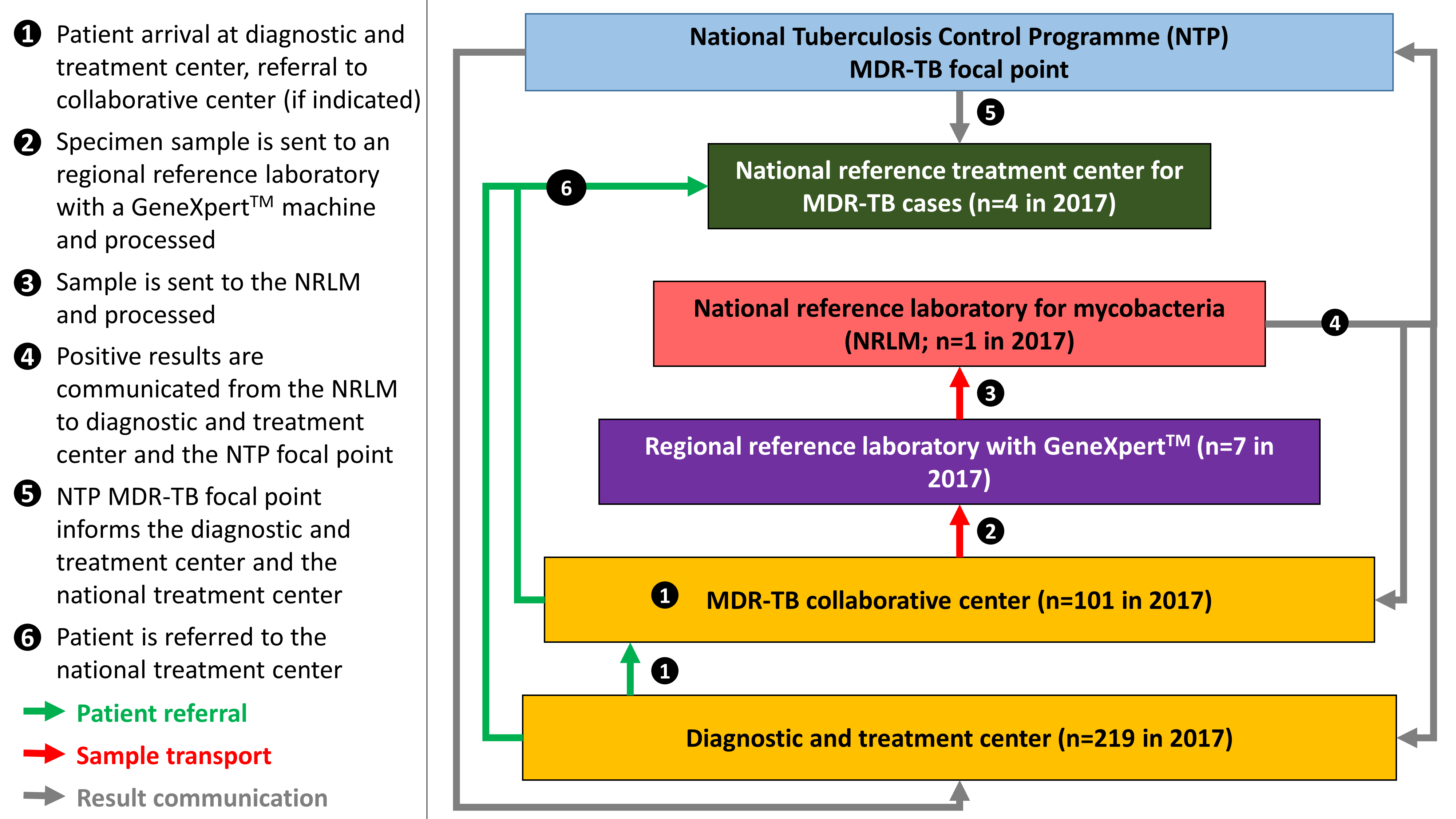

Supplement: Supplementary file 4 — Additional file 4. Fig. S4: Infrastructures and processes, MDR-TB surveillance programme, Madagascar, 2012–2017. [file 12916_2020_1626_MOESM4_ESM.tif]
