# Supplementary material for: Multidrug-resistant tuberculosis surveillance and cascade of care in Madagascar: a five-year (2012–2017) retrospective study
Source: BMC Med. 2020 Jun 30;18:173. doi: 10.1186/s12916-020-01626-6 (PMC7325144; doi:10.1186/s12916-020-01626-6)
Supplement: Supplementary file 3 — Additional file 3. Appendix S3: Calculation of steps and gaps for the TB and MDR-TB cascade of care, Madagascar, 2017. [file 12916_2020_1626_MOESM3_ESM.docx]

**Additional file 3: Appendix S3: Calculation of steps and gaps for the TB and MDR-TB cascade of care, Madagascar, 2017**

# Step 1: Number of prevalent TB all forms, presumptive MDR-TB and MDR-TB cases

## Step 1-A Overall cascade (all forms of TB)

**Overall prevalence of TB all forms based on the WHO estimate (1) = 61,880 (95% CI: 40,040-88,400)**

## Step 1-B Overall cascade (presumptive MDR-TB)

Overall prevalence of presumptive MDR-TB is calculated by multiplying the overall prevalence of TB all forms by the proportion of notified TB patients with presumptive MDR-TB (i.e. disease relapse, treatment failure, retreatment patients, confirmed MDR-TB contact) at country level.

*Assumption 1: The total number of prevalent TB patients per WHO estimates (1) (step 1-A) = 61,880 (95% CI: 40,040-88,400)*

*Assumption 2: From the Madagascar trimestral reports on TB case notifications and clinical forms – 2017 (2), the total number of notified TB all forms patients = 31,618*

*Assumption 3: From the Madagascar trimestral reports on TB case notifications and clinical forms – 2017 (2), the total number of notified presumptive MDR-TB patients = 1,863*

*Assumption 4: The proportion of presumptive MDR-TB patients notified patients among TB all forms patients = 1,863 / 31,618 = 0.0589 (95% CI: 0.0564 – 0.0615)*

*Assumption 5: The proportion of notified presumptive MDR-TB patients among notified TB all forms patients is the same as the proportion of presumptive MDR-TB patients among all TB all forms patients.*

*Assumption 6: The total number of presumptive MDR-TB patients = (61,880) (0.0589) = 3,646*

**Overall prevalence of presumptive MDR-TB estimate = 3,646 (95% CI: 2,258 – 5,437)**

## Step 1-C Overall cascade (MDR-TB)

Overall prevalence of MDR-TB is calculated by multiplying the overall prevalence of MDR/RR-TB as assessed by WHO (1) by the proportion of MDR-TB patients among all RR-TB over the 5-year retrospective period.

*Assumption 7: The total number of TB (MDR/RR-TB) based on the 2018 WHO estimates (1) = 442 (95% CI: 88-1,092)*

*Assumption 8: The total number of DST-confirmed MDR-TB from the Madagascar National Tuberculosis Program Reference Laboratory Data on TB Diagnosis and Drug Resistance Testing – 2017 (3) and 2012-2017 period = 98*

*Assumption 9: The total number of DST-confirmed RR-TB from the Madagascar National Tuberculosis Program Reference Laboratory Data on TB Diagnosis and Drug Resistance Testing – 2017 (3) and 2012-2017 period = 127*

*Assumption 10: The proportion of MDR-TB among all MDR/RR-TB from the Madagascar National Tuberculosis Program Reference Laboratory Data on TB Diagnosis and Drug Resistance Testing – 2017 (3) and 2012-2017 period = 98 / 127 = 0.7717 (95% CI: 0.6909 – 0.8365)*

*Assumption 11: The proportion of MDR-TB among all MDR/RR-TB from the Madagascar National Tuberculosis Program Reference Laboratory Data on TB Diagnosis and Drug Resistance Testing is the same as the proportion of MDR-TB among all MDR/RR-TB.*

*Assumption 10: The total number MDR-TB = (442) (0.7717) = 341*

**Overall prevalence of MDR-TB estimate = 341 (95% CI: 61 – 913)**

*Collateral validation data 1:*

*According to the first and most recent national survey on TB drug resistance which dates from 2005-06, 0.2% (95% CI: 0.0 - 0.5) of new TB patients and 3.4% (95% CI: 0.00 - 7.28) were MDR-TB patients (4). Applying those rates of MDR-TB to the total number of TB notifications yields 239 (95% CI: 155 - 343)*

*Limitation 1:*

*Modeled data from the national survey on TB drug resistance suggest a lower number of MDR-TB patients with a narrower CI than more recent WHO estimates. Following discussion with the NTP and investigators, given the expected continuous increase in MDR-TB rates, the outdated nature and smaller sampling size of the national survey (total n=1,275), it is believed that the WHO-based and better powered (total n=2,391) laboratory recent estimates are more accurate. Nevertheless, estimations based on the 2005-06 DST survey were thought to support the plausibility of the presented numbers.*

# Gap 1: Number of TB all forms, presumptive MDR-TB or MDR-TB cases who do not reach TB diagnostic and treatment centers

## Gap 1-A Overall cascade (all forms of TB)

*Assumption 11: The total number of prevalent TB patients all forms per WHO estimates (step 1-A)* = 61,880 (95% CI: 40,040 - 88,400)

*Assumption 12: The total number of TB all forms patients evaluated within the NTP network of diagnosis and treatment centers (step 2-A) = 51,419 (95%CI: 46,744* - *57,250)*

*Assumption 13: Overall cascade Gap 1-A estimate = Overall cascade Step 1-A estimate – Overall cascade Step 2-A estimate = 61,880 – 51,419 = 10,461*

**Overall cascade Gap 1-A estimate = 10,461 (95% CI: -17,210 – 41,656): 100% - 17% = 83%**

## Gap 1-B Overall cascade (presumptive MDR-TB)

*Assumption 14: The total number of presumptive MDR-TB based on the WHO estimates and primary NTP notification data (step 1-B) = 3,646 (95% CI: 2,258 -5,436)*

*Assumption 15: The total number of presumptive MDR-TB patients evaluated within the NTP network of diagnosis and treatment centers (step 2-B) = 3,563 (95%CI: 3,197 — 4,046)*

*Assumption 16: Overall cascade Gap 1-B estimate = Overall cascade Step 1-B estimate – Overall cascade Step 2 estimate = 3,645 – 3,563 = 82*

**Overall cascade Gap 1-B estimate = 82 (95% CI: -1,788 – 2,239): 100% - 2% = 98%**

## Gap 1-C Overall cascade (MDR-TB)

*Assumption 17: The total number of MDR-TB based on the WHO estimates and national TB reference laboratory data (step 1-C) = 341 (95% CI: 61 - 913)*

*Assumption 18: The total number of MDR-TB patients evaluated within the NTP network of diagnosis and treatment centers (step 2-C) = 224 (95% CI: 170 - 330)*

*Assumption 19Overall cascade Gap 1-C estimate = Overall cascade Step 1-C estimate –Overall cascade Step 2-C estimate = 341 – 224 = 117*

**Overall cascade Gap 1-C estimate = 117 (95% CI: -269 - 743): 100% - 34% = 66%**

# Step 2: Number of TB all forms, presumptive MDR-TB or MDR-TB patients evaluated at TB diagnostic and treatment centers

## Step 2-A Evaluated at TB diagnostic and treatment centers (all forms of TB)

**New smear-positive TB patients**

*Assumption 20: All new patients with suspected pulmonary TB are tested with three consecutive smear microscopy as per NTP Clinical Guidelines (5).*

*Assumption 21: Incremental yield of sputum smear microscopy for diagnosis of smear-positive pulmonary TB is respectively 11.9% for the second sputum and 2.3% for the third sputum (CI not presented) (6)*

*Assumption 22: From the NTP Reference Laboratory Data on TB Diagnosis and Drug Resistance Testing – 2017 (3), 184,320 smear microscopy testing were performed for initial diagnostic testing of 82,111 distinct new patients corresponding to a mean of 2.2448 smear testing per patient.*

*Assumption 23: Patients tested by smear microscopy can be divided as 51,104 patients providing three sputum samples and 31,007 failling to provide second sputum what represents a proportion of 0.3776 (95% CI: 0.3743 – 0.3809)*

*Assumption 24: Proportion of smear-positive patients presenting to TB diagnostic and treatment who remain undiagnosed because of failiure to provide a second sputum smear = 0.119 x 0.3776 (95% CI: 0.3743 – 0.3809) = 0.0449 (95% CI: 0.0445 – 0.0453)*

*Assumption 25: Number of new smear-positive patients diagnosed (Step 3) = 22,480*

*Assumption 26: New smear-positive TB patients evaluated at TB diagnostic and treatment = 22,480 / (1 – 0.0449) = 23,538*

New smear-positive patients evaluated at TB diagnostic and treatment Step 2 estimate = 23,538 (95% CI: 23,430 – 23,651)

**Smear-positive presumptive MDR-TB patients**

*Assumption 27: Number of smear-positive presumptive MDR-TB patients diagnosed (Step 3)* = 1,903

*Assumption 28: Smear-positive presumptive MDR-TB patients evaluated at TB diagnostic and treatment center = 1,903 / (1 – 0.0449) = 1,992*

Smear-positive presumptive MDR-TB patients evaluated at TB diagnostic *and treatment center* Step 2 estimate = 1,992 (95% CI: 1,962 – 2,028)

**New smear-negative TB patients**

*Assumption 29: In a recent Malagasy study evaluating the performance and implementation of molecular and microscopy assays for TB diagnosis which recruited an unbiased population of 517 TB presumptive patients, culture diagnosed 148 fluorescence smear microscopy positive and 53 fluorescence smear microscopy negative patients. The sensitivity of fluorescence microscopy testing was found to be 73.6 % (95% CI: 67.1 – 79.3) (7).*

*Assumption 30: Fluorescence microscopy is on average 10% more sensitive than conventional microscopy (8).*

*Assumption 31: The sensitivity of conventional microscopy in Madagascar = 73.6% - 10.0% = 63.6% (95% CI: 56.8 – 70.0).CI calculated by modeling microscopy smear-positive and smear-negative proportions in the cited study population.*

*Collateral validation data 2:*

*The sensitivity of serial testing with smear microscopy in a systematic review is 64% (6). This supports estimations based on the Madagascar study which is used for cascade modelling in this study.*

*Limitation 2:*

*Based on our calculation, the error margin on the sensitivity of conventional microscopy and resulting smear-positive to smear-negative patients ratio is high. This introduces a high margin of uncertainty on this step of the cascade but is believed to accurately reflect Madagascar’s reality where the quality of equipment, consumables, technique and interpretation training for conventional microscopy can be extensively heterogeneous.*

*Assumption 32: The ratio of smear-positive to smear-negative TB patients = 128:73 = 1.75:1*

*Assumption 33: The ratio of smear-positive to smear-negative TB patients lower bound = 114:87 = 1.32:1*

*Assumption 34: The ratio of smear-positive to smear-negative TB patients upper bound = 141:60 = 2.34:1*

*Assumption 35: the number of new smear-positive patients evaluated at TB diagnostic and treatment centers Step 2 estimate = 23,538 (95% CI: 23,430 – 23,651)*

New smear-negative TB patients evaluated at TB diagnostic and treatment centers Step 2 estimate = 23,538 / 1.75 = 13,425 (95% CI: 10,032 – 17,966)

**Smear-negative presumptive MDR-TB patients**

*Assumption 36: the number of smear-positive presumptive MDR-TB patients evaluated at TB diagnostic and treatment centers Step 2 estimate = 1,992 (95% CI: 1,992 – 1,993)*

Smear-negative presumptive MDR-TB patients evaluated at TB diagnostic *and treatment centers* Step 2 estimate = 1,992 / 1.75 = 1,136 (95% CI: 840 – 1541)

**Extrapulmonary TB patients (new and presumptive MDR-TB)**

*Assumption 37: The proportion of extrapulmonary TB patients who remain undiagnosed despite being evaluated at TB diagnostic and treatment centers (Gap 2) is equivalent to the average of the proportion of undiagnosed smear-positive TB patients (Gap 2) and the proportion of undiagnosed smear-negative TB patients (Gap 2).*

*Assumption 38: Proportion of smear-positive patients presenting to TB diagnostic and treatment centers who remain undiagnosed = 0.0449 (95% CI: 0.0445 – 0.0453)*

*Assumption 39: Proportion of smear-negative patients presenting to TB diagnostic and treatment centers who remain undiagnosed estimate (95% CI: lower bound and upper bound) = (Smear-negative Step 2 – Smear negative Step 3) / Smear-negative step 2 = (13,425 +1,136 - 3,679 - 315) / (13,425 + 1,136) = 0.7257 (95% CI: 0.6365 – 0.7926)*

*Assumption 40: The proportion of undiagnosed extrapulmonary TB patients = the mean of the proportion of undiagnosed smear-positive patients and the proportion of undiagnosed smear-negative TB patients = 0.3853 (95% CI: 0.3405 – 0.4190)*

*Assumption 41: the number of extrapulmonary TB patients (new TB patients and presumptive MDR-TB patients) diagnosed (Step 3) estimate = 6,963 (95% CI: 6,911 – 7,010)*

*Assumption 42:* Extrapulmonary TB Step 2 estimates = 6,963 / (1 – 0.3853) = 11,327

Extrapulmonary TB Step 2 estimates = 11,327 (95% CI: 10,480 – 12,064)

**Overall**

**Overall combined Step 2 estimates for all forms of TB = 51,419 (95% CI: 46,744 – 57,250)**

## Step 2-B Evaluated at TB diagnostic and treatment centers (presumptive MDR-TB)

**Extrapulmonary presumptive MDR-TB patients**

*Assumption 43: the number of extrapulmonary presumptive MDR-TB patients diagnosed (Step 3) estimate = 267 (95% CI: 260 – 277)*

*Assumption 44: Extrapulmonary presumptive MDR-TB patients Step 2 estimates = 267 / (1 – 0.3853) = 434*

Extrapulmonary *presumptive MDR-TB patients* Step 2 estimates = 434 (95% CI: 395 – 478)

**Overall**

**Overall combined Step 2 estimates for presumptive MDR-TB patients = 3,563 (95% CI: 3,197 – 4,046)**

## Step 2-C Evaluated at TB diagnostic and treatment centers (MDR-TB)

*Assumption 45: The total number of MDR-TB patients evaluated at TB diagnostic and treatment centers is the sum of the number of MDR-TB patients diagnosed (Step 3-C), those appropriately tested but missed because of imperfect confirmation assay sensitivity (GeneXpert), those appropriately tested but missed because of imperfect screening assay sensitivity (smear microscopy) and those assessed for TB but unrecognized clinically as presumptive MDR-TB patients.*

*Assumption 46: From the NTP MDR clinical management data (9), the total number of diagnosed MDR-TB patients (Step 3) = 24 (95% CI: 22 – 27)*

*Assumption 47: All new patients with clinically suspected of presumptive MDR-TB are screened with smear microscopy as per Madagascar NTP Clinical Guidelines (5).*

*Assumption 48: The sensitivity of conventional microscopy in Madagascar is 63.6% (95% CI: 56.8 – 70.0).*

*Assumption 49: The total number of MDR-TB patients tested for TB but missed because of imperfect smear microscopy sensitivity = 24 / (0.636) - 24 = 13 (95% CI: 10 – 20)*

*Assumption 50: All smear-positive patients with presumptive MDR-TB are tested with GeneXpert MTB/RIF for RIF resistance confirmation as per NTP Clinical Guidelines (5).*

*Assumption 51: GeneXpert MTB/RIF sensitivity for RIF resistance is 95% ( 95% CI: 90% - 97%) (10)*

*Assumption 52: The total number of MDR-TB patients tested for resistance but missed because of imperfect sensitivity = 24 / (0.95) - 24 = 1 (95% CI: 1 – 3)*

*Assumption 53: From the Madagascar NTP Reference Laboratory Data on TB Diagnosis and Drug Resistance Testing – 2017 (3) a total of 610 presumptive MDR-TB patients were tested for MDR-TB.*

*Assumption 54: Total presumptive MDR-TB patients evaluated at TB diagnostic and treatment centers = 3,563 (95% CI: 3,197 – 4,046)*

*Assumption 55: The rate of MDR-TB patient among presumptive MDR-TB patients evaluated at TB diagnostic and treatment centers and appropriately tested (diagnosed or missed) for MDR-TB is the same as the rate of MDR-TB patient among presumptive MDR-TB patients evaluated at TB diagnostic and treatment centers not tested for MDR-TB.*

*Assumption 56: The total number of MDR-TB patient evaluated at TB diagnostic and treatment centers = (3,563) (24 +1 + 13) / (610) = 224*

*Limitation 3: A significant gap in the MDR-TB cascade results from the use of smear microscopy screening of presumptive MDR-TB patients. Although some patients are referedl to the MDR TB program when testing smear positive despite a high pre-test clinical suspicion, this represents a minority (5.4%) of the samples referred. Given the inconsistency in the referral indications, for the cascade of care calculation purposes, we assume that referral is contingent on testing positive on smear microscopy.*

**Total number of MDR-TB patient evaluated at TB diagnostic *and treatment centers* – Step 2 – estimate = 224 (95% CI: 170 – 330)**

# Gap 2: Number of TB all forms, presumptive MDR-TB or MDR-TB evaluated at TB diagnostic and treatment centers who remain undiagnosed

## Gap 2-A Overall cascade (all forms of TB)

*Assumption 57: The total number of patients evaluated for all forms of TB within the NTP network of diagnosis and treatment centers (step 2-A) = 51,419 (95%CI: 46,744 — 57,250)*

*Assumption 58: The total number of diagnosed all forms of TB patients per WHO estimates (step 3-A)* = 35,339 (95% CI: 35,124 - 35,570)

*Assumption 59: Overall cascade Gap 2-A estimate = Overall cascade Step 2-A estimate – Overall cascade Step 3-A estimate = 51,419 – 35,339 = 16,080*

**Overall cascade Gap 2-A estimate = 16,080 (95% CI: 11,174 – 22,126): 100% - 43% = 57%**

## Gap 2-B Overall cascade (presumptive MDR-TB)

*Assumption 60: The total number of patients evaluated for presumptive MDR- TB within the NTP network of diagnosis and treatment centers (step 2-B) = 3,563 (95%CI: 3,197 — 4,046)*

*Assumption 61: The total number of diagnosed presumptive MDR-TB per WHO estimates (step 3-B)* = 2,485 (95% CI: 2,443 – 2,540)

*Assumption 62: Overall cascade Gap 2-B estimate = Overall cascade Step 2-A estimate – Overall cascade Step 3-B estimate = 3,563 – 2,485 = 1,078*

**Overall cascade Gap 2-B estimate = 1,078 (95% CI: 657 – 1,603): 100% - 32% = 68%**

## Gap 2-C Overall cascade (MDR-TB)

*Assumption 63: The total number of MDR-TB patients evaluated within the NTP network of diagnosis and treatment centers (step 2-C) = 224 (95%CI: 170 — 330)*

*Assumption 64: The total number of MDR-TB diagnosed TB per Madagascar NTP Reference Laboratory Data on TB Diagnosis and Drug Resistance Testing – 2017 (3)* = 24 (95% CI: 22 – 27)

*Assumption 65: Overall cascade Gap 2-C estimate = Overall cascade Step 2-A estimate – Overall cascade Step 2-B estimate = 224 – 24 = 200*

**Overall cascade Gap 2-C estimate = 200 (95% CI: 143 – 308): 100% - 93% = 7%**

# Step 3: Number of patients successfully diagnosed with TB all forms, presumptive MDR-TB or MDR-TB at TB diagnostic and treatment centers

## Step 3-A Successfully diagnosed (all forms of TB)

**New smear-positive TB patients**

*Assumption 67: From the Madagascar trimestral report on TB therapy and clinical management data – 2017 (11) and a five-year retrospective review of country clinical management data the total pre/per-treatment loss to follow-up rate for new smear-positive TB patients = 0.0883 (95% CI: 0.0845 – 0.0923)*

*Assumption 68: The loss to follow-up rate is constant throughout the cascade of care steps (i.e. pre-/per-treatment)*

*Assumption 69: From the Madagascar trimestral report on TB therapy clinical management data – 2017 (11), the number of new smear-positive TB patients registered for treatment (step 4) = 20,495*

*Assumption 70: The total number of new smear-positive TB patients diagnosed with TB = 20,495 / (1 - 0.0883) = 22,480*

New smear-positive step 3 estimate = 22,480 (95% CI: 22,387 – 22,579)

*Collateral validation data 3:*

*From the Madagascar NTP Reference Laboratory Data on TB Diagnosis and Drug Resistance Testing – 2017 (3), the total number of smear-positive patients diagnosed with TB = 21,770*

*Collateral validation data 4:*

*From the Madagascar trimestral report on TB therapy clinical management data – 2017 (11), the number of new smear-positive TB patients registered for treatment (step 4) = 20,495*

*From the Madagascar trimestral report on TB therapy and clinical management data – 2017 (11), the number of smear-positive presumptive MDR-TB patients registered for treatment (step 4) = 1,708*

*The proportion of new smear-positive TB patients registered for treatment among all smear-positive patients registered for treatment = 20,495 / (20,495 + 1,708) = 20,495 / 22,203 = 0.9231(95% CI: 0.9195 - 0.9265)*

*The proportion of new smear-positive patients diagnosed with TB among all smear-positive patients diagnosed with TB is the same as the proportion of new smear-positive TB patients registered for treatment among all smear-positive patients registered for treatment.*

*Laboratory and treatment registration derived new smear-positive patients Step 3 estimate = (21,770) (0.9231) = 20,095*

*Collateral validation data 5:*

*From the Madagascar trimestral reports on TB case notifications and clinical forms – 2017 (2), the number of new smear-positive TB patients notified = 20,192.*

**Smear-positive presumptive MDR-TB patients**

*Assumption 71: From the Madagascar trimestral report on TB therapy and clinical management data – 2017 (11) and a five-year retrospective review of country clinical management data the total pre/per-treatment loss to follow-up rate for presumptive MDR-TB patients = 0.1025 (95% CI: 0.0889 – 0.1178)*

*Assumption 72: The loss to follow-up rate is constant throughout the cascade of care steps (i.e. pre-/per-treatment)*

*Assumption 73: From the Madagascar trimestral report on TB therapy clinical management data – 2017 (11), the number of smear-positive presumptive TB patients registered for treatment (step 4) = 1,708*

*Assumption 74: The total number of smear-positive presumptive MDR-TB patients diagnosed with TB = 1,708 / (1 - 0.1025) = 1,903*

Smear-positive presumptive MDR-TB patients step 3 estimate = 1,903 (95% CI: 1,875 – 1,936)

*Collateral validation data 6:*

*From the Madagascar trimestral report on TB therapy clinical management data – 2017 (11), the number of new smear-positive TB patients registered for treatment (step 4) = 20,495*

*From the Madagascar trimestral report on TB therapy and clinical management data – 2017 (11), the number of smear-positive presumptive MDR-TB patients registered for treatment (step 4) = 1,708*

*The proportion of smear-positive presumptive MDR-TB patients registered for treatment among all smear-positive patients registered for treatment = 1,708 / (20,495 + 1,708) = 1,708 / 22,203 = 0.0769*

*The proportion of smear-positive presumptive MDR-TB patients diagnosed with TB among all smear-positive patients diagnosed with TB is the same as the proportion of smear-positive presumptive MDR-TB patients registered for treatment among all smear-positive patients registered for treatment.*

*Laboratory and treatment registration derived presumptive MDR-TB patients Step 3 estimate = (21,770) (0.0769) = 1,674*

*Collateral validation data 7:*

*From the Madagascar trimestral reports on TB case notifications and clinical forms – 2017 (2), the number of presumptive MDR-TB patients notified = 1,863.*

**New smear-negative TB patients**

*Assumption 75: From the Madagascar trimestral report on TB therapy and clinical management data – 2017 (11) and a five-year retrospective review of country clinical management data the total pre-/per-treatment loss to follow-up rate for new smear-negative TB patients = 0.0837 (95% CI: 0.0748 – 0.0935)*

*Assumption 76: The loss to follow-up rate is constant throughout the cascade of care steps (i.e. pre-/per-treatment)*

*Assumption 77: From the Madagascar trimestral report on TB therapy and clinical management data – 2017 (11), the number of new smear-negative TB patients registered for treatment (step 4) = 3,371*

*Assumption 78: The total number of new smear-negative TB patients diagnosed with TB = 3,371 / (1 - 0.0837) = 3,679*

New smear-negative step 3 estimate = 3,679 (95% CI: 3,645 – 3,719)

*Collateral validation data 8:*

*Assumption 79: From the Madagascar trimestral reports on TB case notifications and clinical forms – 2017 (2), the number of smear-negative TB patients notified = 2,854. Smear-negative patients notifications data are not disaggregated and thus include new, disease relapse, treatment failure or retreatment patients.*

**Smear-negative presumptive MDR-TB patients**

*Assumption 80: From the Madagascar trimestral report on TB therapy and clinical management data – 2017 (11) and a five-year retrospective review of country clinical management data the total pre/per-treatment loss to follow-up rate for smear-negative presumptive MDR-TB patients = 0.0572 (95% CI: 0.0355 – 0.0903)*

*Assumption 81: The loss to follow-up rate is constant throughout the cascade of care steps (i.e. pre-/per-treatment)*

*Assumption 82: From the Madagascar trimestral report on TB therapy and clinical management data – 2017 (11), the number of smear-negative presumptive MDR-TB patients registered for treatment (step 4) = 297*

*Assumption 83: The total number of smear-negative presumptive MDR-TB patients diagnosed with TB = 297 / (1 – 0.0572) = 315*

Smear-negative presumptive MDR-TB patients Step 3 estimate = 315 (95% CI: 308 – 326)

*Collateral validation data 9:*

*From the Madagascar trimestral reports on TB case notifications and clinical forms – 2017 (2), the number of smear-negative TB patients notified = 2,854. Smear-negative patients notifications data are not disaggregated and thus include new, disease relapse, treatment failure or retreatment patients.*

**New** **extrapulmonary**

*Assumption 84: From the Madagascar trimestral report on TB therapy and clinical management data – 2017 (11) and a five-year retrospective review of country clinical management data the total pre/per-treatment loss to follow-up rate for new extrapulmonary patients = 0.0575 (95% CI: 0.0521 – 0.0636)*

*Assumption 85: The loss to follow-up rate is constant throughout the cascade of care steps (i.e. pre-/per-treatment)*

*Assumption 86: From the Madagascar trimestral report on TB therapy and clinical management data – 2017 (11), the number of new extrapulmonary TB patients registered for treatment (step 4) = 6,304*

*Assumption 87: The total number of new extrapulmonary TB patients diagnosed with TB = 6,556 / (1 – 0.0575) = 6,696*

Extrapulmonary TB patients Step 3 estimate = 6,696 (95% CI: 6,650 – 6,732)

*Collateral validation data 10:*

*From the Madagascar trimestral reports on TB case notifications and clinical forms – 2017 (2), the number of new extrapulmonary TB patients notified = 6,252.*

**Re-treatment** **extrapulmonary**

*Assumption 88: From the Madagascar trimestral report on TB therapy and clinical management data – 2017 (11) and a five-year retrospective review of country clinical management data the total pre-/per-treatment loss to follow-up rate for re-treatment extrapulmonary patients = 0.0555 (95% CI: 0.0326 – 0.0918)*

*Assumption 89: The loss to follow-up rate is constant throughout the cascade of care steps (i.e. pre-/per-treatment)*

*Assumption 90: From the Madagascar trimestral report on TB therapy and clinical management data – 2017 (11), the number of re-treatment extrapulmonary TB patients registered for treatment (step 4) = 252*

*Assumption 91: The total number of re-treatment extrapulmonary TB patients diagnosed with TB = 6,556 / (1 – 0.0555) = 267*

Re-treatment extrapulmonary TB patients Step 3 estimate = 267 (95% CI: 260 – 277)

*Collateral validation data 11:*

*From the Madagascar trimestral reports on TB case notifications and clinical forms – 2017 (2), the number of re-treatment extrapulmonary TB patients notified = 262*

**Overall**

**Overall combined step 3 estimates for all forms of TB = 35,339 (95% CI: 35,124 – 35,570)**

*Limitation 4:*

*Discrepancy between notification and clinical management data cannot be resolved with available data. Following discussion with the NTP and investigators, it is believed that the clinical management data, accounting for loss to follow-up, is more accurate and that the lower number of new smear-positive TB patients in the laboratory data and notification data is explained by potential missing of primary facility reports or under-recording of patients transferred to treatment facilities.*

## Step 3-B Successfully Diagnosed (presumptive MDR-TB)

**Overall combined step 3 estimates for presumptive MDR-TB = 2,485 (95% CI: 2,443 – 2,540)**

*Limitation 5:*

*Discrepancy between notification and clinical management data cannot be resolved with available data. Following discussion with the NTP and investigators, it is believed that the clinical management data, accounting for loss to follow-up, is more accurate and that the lower number of new smear-positive TB patients in the laboratory data and notification data is explained by potential missing of primary facility reports or under-recording of patients transferred to treatment facilities.*

## Step 3-C Successfully Diagnosed (MDR- TB)

*Assumption 92: From the MDR clinical management data (9) and a five-year retrospective review of country clinical management data the total pre-/per-treatment loss to follow-up rate for MDR-TB patients = 0.1127 (95% CI: 0.0557 – 0.2094)*

*Assumption 93: The loss to follow-up rate is constant throughout the cascade of care steps (i.e. pre-/per-treatment)*

*Assumption 94: From the Madagascar National Tuberculosis Program Reference Laboratory Data on TB Diagnosis and Drug Resistance Testing – 2017 (3), the total number of MDR-TB confirmed on DST = 21*

*Assumption 95: The total number of diagnosed MDR-TB = 21 / (1 - 0.1127) = 24*

**MDR-TB step 3 estimate = 24 (95% CI: 22 – 27)**

*Collateral validation data 12:*

*From the Madagascar MDR clinical management data (9), the number of MDR-TB patients registered for treatment (step 4) = 18*

*Limitation 6:*

*Discrepancy between Madagascar MDR-TB clinical management data and NTP Reference Laboratory Data are explained by previous NTP diagnosis algorithms whereas at the time only culture results were considered for the diagnosis of MDR-TB. In this context the NTP and investigators consider the laboratory data to be more accurate.*

# Gap 3: Number of TB all forms, presumptive MDR-TB or MDR-TB cases diagnosed at TB diagnostic and treatment centers who are not registered for TB treatment (i.e. “pretreatment loss to follow-up” or “initial default”)

## Gap 3-A Overall cascade (all forms of TB)

*Assumption 96: The number of all forms of TB patients diagnosed (Step3) = 35,339 (95% CI: 35,124 – 35,570)*

*Assumption 97: From the Madagascar trimestral report on TB therapy and clinical management data – 2017 (11), the number of all forms of patients registered for treatment = 32,427*

**Cascade Gap 3-A = 2,912 (95% CI: 2,697 – 3,143): 100% - 48% = 52%**

## Gap 3-B Overall cascade (presumptive MDR-TB)

*Assumption 98: The number of presumptive MDR-TB patients diagnosed (Step3) = 2,485 (95% CI: 2,443 – 2,540)*

*Assumption 99: From the Madagascar trimestral report on TB therapy and clinical management data – 2017 (11), the number of presumptive MDR-TB patients registered for treatment = 2,257*

**Cascade Gap 3-B = 228 (95% CI: 186 – 283): 100% - 38% = 62%**

## Gap 3-C Overall cascade (MDR-TB)

*Assumption 100: The number of MDR-TB patients diagnosed (Step3) = 24 (95% CI: 22 – 27)*

*Assumption 101: From the Madagascar trimestral report on TB therapy and clinical management data – 2017 (11), the number of MDR-TB patients registered for treatment = 18*

**Cascade Gap 3-C = 6 (95% CI: 4 – 9): 100% - 95% = 5%**

# Step 4: Number of TB all forms, presumptive MDR-TB or MDR-TB patients registered for TB treatment

The numbers of patients registered in TB treatment for all forms of TB (new smear-positive, presumptive MDR-TB smear-positive, new smear-negative, presumptive MDR-TB smear-negative, new extrapulmonary, presumptive MDR-TB extra-pulmonary) were extracted from the Madagascar trimestral report on TB therapy and clinical management data – 2017 (11)

## Step 4-A Registered for TB treatment (all forms of TB)

**New smear-positive TB patientss**

New smear-positive Step 4 estimates from the Madagascar trimestral report on TB therapy and clinical management data – 2017 (11) = 20,495

**Smear-positive presumptive MDR-TB patients**

Smear-positive presumptive MDR-TB Step 4 estimates from the Madagascar trimestral report on TB therapy and clinical management data – 2017 (11) = 1,708

**New smear-negative TB patients**

New smear-negative Step 4 estimates from the Madagascar trimestral report on TB therapy and clinical management data – 2017 (11) = 3,371

**Smear negative presumptive MDR-TB patients**

Smear negative presumptive MDR-TB Step 4 estimates from the Madagascar trimestral report on TB therapy and clinical management data – 2017 (11) = 297

**New extrapulmonary TB patients**

New extrapulmonary TB Step 4 estimates from the Madagascar trimestral report on TB therapy and clinical management data – 2017 (11) = 6,304

**Extrapulmonary presumptive MDR-TB patients**

Extrapulmonary presumptive MDR-TB Step 4 estimates from the Madagascar trimestral report on TB therapy and clinical management data – 2017 (11) = 252

**Overall**

**Overall Step 4 estimates (TB all forms) = 32,427**

## Step 4-B Registered for TB treatment (presumptive MDR-TB)

**Overall**

**Overall Step 4 estimates (presumptive MDR-TB) = 2,257**

## Step 4-C Registered for TB treatment (MDR- TB)

**MDR-TB Step 4 estimates from the Madagascar trimestral report on TB therapy and clinical management data – 2017 (11) = 18**

# Gap 4: number of TB all forms, presumptive MDR-TB and MDR-TB patients who fail therapy, are lost to follow-up, or die during TB treatment

## Gap 4-A Overall cascade (all forms of TB)

*Assumption 102: From the Madagascar trimestral report on TB therapy and clinical management data – 2017 (11), the number of all forms of TB patients registered for treatment = 32,427*

*Assumption 103: From the Madagascar trimestral report on TB therapy and clinical management data – 2017 (11), the number of all forms of TB patients reaching therapy completion or cure = 28,178*

**Cascade Gap 3-A = 4,249 (no confidence interval as both values are extracted from *the Madagascar trimestral report on TB therapy and clinical management data – 2017 (11)):* 100% - 54% = 46%**

## Gap 4-B Overall cascade (presumptive MDR-TB)

*Assumption 104: From the Madagascar trimestral report on TB therapy and clinical management data – 2017 (11), the number of presumptive MDR-TB patients registered for treatment = 2,257*

*Assumption 105: From the Madagascar trimestral report on TB therapy and clinical management data – 2017 (11), the number of presumptive MDR-TB patients reaching therapy completion or cure = 1,852*

**Cascade Gap 3-B = 405 (no confidence interval as both values are extracted from *the Madagascar trimestral report on TB therapy and clinical management data – 2017 (11)):* 100% - 49% = 51%**

## Gap 4-C Overall cascade (MDR-TB)

*Assumption 106: From the Madagascar trimestral report on TB therapy and clinical management data – 2017 (11), the number of MDR-TB patients registered for treatment = 18*

*Assumption 107: From the Madagascar trimestral report on TB therapy and clinical management data – 2017 (11), the number of MDR-TB patients reaching therapy completion or cure (Step 5-C) = 8*

**Cascade Gap 3-B = 10 (no confidence interval as both values are extracted from *the Madagascar trimestral report on TB therapy and clinical management data – 2017 (11)):* 100% - 96% = 4%**

# Step 5: Number of TB all forms, presumptive MDR-TB and MDR-TB patients who achieve treatment completion or cure

The numbers of patients who achieved treatment completion or cure for all forms of TB (new smear-positive, presumptive MDR-TB smear-positive, new smear-negative, presumptive MDR-TB, new extrapulmonary TB, presumptive MDR-TB extrapulmonary) were extracted from the Madagascar trimestral report on TB therapy and clinical management data – 2017 (11)

## Step 5-A Completed treatment or cured (all forms of TB)

**New smear-positive TB patients**

New smear-positive Step 5 estimates from the Madagascar trimestral report on TB therapy and clinical management data – 2017 (11) = 2,569

**Smear-positive presumptive MDR-TB patients**

Smear-positive presumptive MDR-TB Step 5 estimates from the Madagascar trimestral report on TB therapy and clinical management data – 2017 (11) = 232

**New smear-negative TB patients**

New smear-negative Step 5 estimates from the Madagascar trimestral report on TB therapy and clinical management data – 2017 (11) = 2,919

**Smear negative presumptive MDR-TB patients**

Smear negative presumptive MDR-TB patients Step 5 estimates from the Madagascar trimestral report on TB therapy and clinical management data – 2017 (11) = 253

**New extrapulmonary TB**

New extrapulmonary TB Step 5 estimates from the Madagascar trimestral report on TB therapy and clinical management data – 2017 (11) = 5,602

**Presumptive MDR-TB extrapulmonary patients**

Extrapulmonary presumptive MDR-TB Step 5 estimates from the Madagascar trimestral report on TB therapy and clinical management data – 2017 (11) = 225

**Overall**

**Overall Step 5 estimates (TB all forms) = 28,178**

## Step 5-B Completed treatment or cured (presumptive MDR-TB)

**Overall**

**Overall Step 5 estimates (TB all forms) = 1,852**

## Step 5-C Completed treatment or cured (MDR- TB)

**MDR-TB Step 5 estimates from the Madagascar trimestral report on TB therapy and clinical management data – 2017 (11) = 8**

# Gap 5: Number of TB all forms, presumptive MDR-TB and MDR-TB patients who experience death or TB recurrence in the first 12-24 months after completing therapy

## Gap 5-A Overall cascade (all forms of TB)

*Assumption 108: From the Madagascar trimestral report on TB therapy and clinical management data – 2017 (11), the number of all forms of TB patients reaching therapy completion or cure = 28,178*

*Assumption 109: Total number of all forms of TB patients alive and recurrence-free 12-24 months after completing therapy (Step 6) =* 27,996 (95% CI: 27,774 – 28,108)

**Cascade Gap 5-A = 28,178 – 27,996 = 182 (95% CI: 70 – 404): 100% - 55% = 45%**

## Gap 5-B Overall cascade (presumptive MDR-TB)

*Assumption 110: From the Madagascar trimestral report on TB therapy and clinical management data – 2017 (11), the number of presumptive MDR-TB patients reaching therapy completion or cure = 1,852*

*Assumption 111: Total number of presumptive MDR-TB patients alive and recurrence-free 12-24 months after completing therapy (Step 6) =* 1,670 *(95% CI: 1,448 – 1,782)*

**Cascade Gap 5-B = 1,852 – 1,670 = 182 (95% CI: 70 – 404): 100% - 54% = 46%**

## Gap 5-C Overall cascade (MDR-TB)

**Cascade Gap 5-C = 8 – 8 = 0 (no confidence interval as both values are extracted from *the Madagascar trimestral report on TB therapy and clinical management data – 2017 (11)):* 100% - 98% = 2%**

# Step 6: Number of TB all forms, presumptive MDR-TB and MDR-TB patients who are alive and TB recurrence-free 12-24 months after completing therapy

## Step 6-A Alive and recurrence-free 12-24 months (all forms of TB)

*Assumption 112: The ratio of relapse occurring before 12 months is 0.9194 (95% CI: 0.8208 – 0.9690) (12).*

*Assumption 113: Total number of all forms of TB patients achieving treatment completion or cure (Step 5) = 28,178*

*Assumption 114: From the Madagascar trimestral report on TB therapy and clinical management data – 2017 (11), total number of relapse or retreatment patients = 2,257*

*Assumption 115: The total number of patient relapsing after 12 months = 2,257 * (1 – 0.9294) = 182 (95% CI: 70 – 404)*

*Assumption 116: Number of TB patients who are alive and TB recurrence-free 12-24 months after completion therapy = 28,178 – 182 = 27,996*

**Number of TB patients who are alive and TB recurrence-free 12-24 months after completion therapy = 27,996 (95% CI: 27,774 – 28,108)**

## Step 6-B Alive and recurrence-free 12-24 months (presumptive MDR-TB)

*Assumption 117: Total number of patients achieving treatment completion or cure (Step 5) = 1,852*

*Assumption 118: Number of presumptive MDR-TB patients who are alive and TB recurrence-free 12-24 months after completion therapy = 1,852 – 182 = 1,670*

**Number of TB patients who are alive and TB recurrence-free 12-24 months after completion therapy = 1,670 *(95% CI: 1,448 – 1,782)***

## Step 6-C Alive and recurrence-free 12-24 months (MDR- TB)

*Assumption 119: MDR-TB patients confirmed cured or having completed therapy are alive and recurrence-free 12-24 months following treatment completion.*

*From the Madagascar trimestral report on TB therapy and clinical management data – 2017 (11) MDR-TB patients confirmed cured = 8*

**MDR-TB patients confirmed cured and recurrence-free 12-24 months following treatment completion = 8**

# References

1. WHO. Madagascar tuberculosis profile. Geneva: World Health Organization; 2017.

2. NTP. Trimestrial reports on TB case notifications and clinical forms - 2017. Antananarivo: Direction de Lutte Contre la Tuberculose; 2017.

3. NTP. National Tuberculosis Program Reference Laboratory Data on TB Diagnosis and Drug Resistance Testing - 2017. Antananarivo: Direction de Lutte Contre la tuberculose 2017.

4. Ramarokoto H, Ratsirahonana O, Soares JL, Ravaosolo J, Ravololonandriana P, Rakotoarisaonina A, et al. First national survey of Mycobacterium tuberculosis drug resistance, Madagascar, 2005-2006. Int J Tuberc Lung Dis. 2010;14(6):745-50.

5. NTP. Manuel du programme national de lutte contre la tuberculose: 5ème édition. Antananarivo: Ministère de la Santé Publique, Sécretariat Général, Direction Générale de la Santé, Direction de Lutte Contre la Tuberculose; 2013.

6. Mase SR, Ramsay A, Ng V, Henry M, Hopewell PC, Cunningham J, et al. Yield of serial sputum specimen examinations in the diagnosis of pulmonary tuberculosis: a systematic review. Int J Tuberc Lung Dis. 2007;11(5):485-95.

7. Rakotosamimanana N, Lapierre SG, Raharimanga V, Raherison MS, Knoblauch AM, Raherinandrasana AH, et al. Performance and impact of GeneXpert MTB/RIF(R) and Loopamp MTBC Detection Kit(R) assays on tuberculosis case detection in Madagascar. BMC Infect Dis. 2019;19(1):542.

8. Steingart KR, Henry M, Ng V, Hopewell PC, Ramsay A, Cunningham J, et al. Fluorescence versus conventional sputum smear microscopy for tuberculosis: a systematic review. Lancet Infect Dis. 2006;6(9):570-81.

9. NTP. MDR clinical management data registry. Antananarivo: Programme de Lutte Contre la Tuberculose; 2017.

10. Steingart KR, Schiller I, Horne DJ, Pai M, Boehme CC, Dendukuri N. Xpert(R) MTB/RIF assay for pulmonary tuberculosis and rifampicin resistance in adults. Cochrane Database Syst Rev. 2014(1):CD009593.

11. NTP. Trimestrial report on TB therapy and clinical management data - 2017. Antananarivo Direction de Lutte Contre la Tuberculose; 2017.

12. Thomas A, Gopi PG, Santha T, Chandrasekaran V, Subramani R, Selvakumar N, et al. Predictors of relapse among pulmonary tuberculosis patients treated in a DOTS programme in South India. Int J Tuberc Lung Dis. 2005;9(5):556-61.
